# Proteomic analysis of *APOE*ε4 carriers implicates lipid metabolism, complement and lymphocyte signaling in cognitive resilience

**DOI:** 10.1186/s13024-024-00772-2

**Published:** 2024-10-31

**Authors:** Keenan A. Walker, Yang An, Abhay Moghekar, Ruin Moaddel, Michael R. Duggan, Zhongsheng Peng, Qu Tian, Luke C. Pilling, Shannon M. Drouin, Mark A. Espeland, Stephen R Rapp, Kathleen M Hayden, Aladdin H. Shadyab, Ramon Casanova, Madhav Thambisetty, Peter R. Rapp, Dimitrios Kapogiannis, Luigi Ferrucci, Susan M. Resnick

**Affiliations:** 1 Laboratory of Behavioral Neuroscience, National Institute on Aging, Baltimore, MD USA; 2 Department of Neurology, Johns Hopkins University School of Medicine, Baltimore, MD USA; 3 Translational Gerontology Branch, National Institute on Aging, Baltimore, MD USA; 4 Department of Clinical & Biomedical Sciences, Faculty of Health & Life Science, University of Exeter, Exeter, UK; 5 Department of Internal Medicine, Wake Forest University School of Medicine, Winston-Salem, NC USA; 6 Department of Biostatistics and Data Science, Wake Forest University School of Medicine, Winston-Salem, NC USA; 7 Department of Psychiatry & Behavioral Medicine, Wake Forest University School of Medicine, Winston-Salem, NC USA; 8 Department of Social Science & Health Policy, Wake Forest University School of Medicine, Winston-Salem, NC USA; 9 Division of Geriatrics, Gerontology, and Palliative Care, Department of Medicine, and Herbert Wertheim School of Public Health and Human Longevity Science, University of California, San Diego, La Jolla, CA USA; 10 Laboratory of Clinical Investigation, National Institute on Aging, Baltimore, MD USA

**Keywords:** Alzheimer’s disease, APOE, Resilience, Cognition, Proteomics, Immunity, Lipids

## Abstract

**Background:**

Apolipoprotein E (*APOE*) ε4 allele is the strongest genetic risk factor for late onset Alzheimer’s disease (AD). This case-cohort study used targeted plasma biomarkers and large-scale proteomics to examine the biological mechanisms that allow some *APOE*ε4 carriers to maintain normal cognitive functioning in older adulthood.

**Methods:**

*APOE*ε4 carriers and *APOE*ε3 homozygotes enrolled in the Women’s Health Initiative Memory Study (WHIMS) from 1996 to 1999 were classified as *resilient* if they remained cognitively unimpaired beyond age 80, and as *non-resilient* if they developed cognitive impairment before or at age 80. AD pathology (Aß_42/40_) and neurodegeneration (NfL, tau) biomarkers, as well as 1007 proteins (Olink) were quantified in blood collected at study enrollment (on average 14 years prior) when participants were cognitively normal. We identified plasma proteins that distinguished between resilient and non-resilient *APOE*ε4 carriers, examined whether these associations generalized to *APOE*ε3 homozygotes, and replicated these findings in the UK Biobank.

**Results:**

A total of 1610 participants were included (baseline age: 71.3 [3.8 SD] years; all White; 42% *APOEε*4 carriers). Compared to resilient *APOE*ε4 carriers, non-resilient *APOE*ε4 carriers had lower Aß_42/40_/tau ratio and greater NfL at baseline. Proteomic analyses identified four proteins differentially expressed between resilient and non-resilient *APOEε*4 carriers at an FDR-corrected *P* < 0.05. While one of the candidate proteins, a marker of neuronal injury (NfL), also distinguished resilient from non-resilient *APOE*ε3 homozygotes, the other three proteins, known to be involved in lipid metabolism (ANGPTL4) and immune signaling (PTX3, NCR1), only predicted resilient vs. non-resilient status among *APOE*ε4 carriers (protein*genotype interaction-*P* < 0.05). Three of these four proteins also predicted 14-year dementia risk among *APOE*ε4 carriers in the UK Biobank validation sample (*N* = 9420). While the candidate proteins showed little to no association with targeted biomarkers of AD pathology, protein network and enrichment analyses suggested that natural killer (NK) cell and T lymphocyte signaling (via PKC-θ) distinguished resilient from non-resilient *APOE*ε4 carriers.

**Conclusions:**

We identified and replicated a plasma proteomic signature associated with cognitive resilience among *APOEε*4 carriers. These proteins implicate specific immune processes in the preservation of cognitive status despite elevated genetic risk for AD. Future studies in diverse cohorts will be needed to assess the generalizability of these results.

**Supplementary Information:**

The online version contains supplementary material available at 10.1186/s13024-024-00772-2.

## Introduction

Alzheimer’s disease (AD) dementia is the most common form of dementia, affecting approximately 6.7 million Americans [1]. Approximately two-thirds of those affected by AD are women [1, 2]. The Apolipoprotein E (*APOE*) ε4 allele is the strongest genetic risk factor for late onset AD, conferring a 3–4 fold increase in risk for AD in population-based samples [3, 4]. The ε4 allele is one of 3 common alleles at the *APOE* locus, with ε2 and ε3 comprising the other alleles. In contrast to the ε4 allele, the *APOE*ε2 allele is associated with decreased risk for AD and greater longevity [5], and the ε3/3 allele is considered the neutral allele with respect to AD risk [6]. *APOE* genotype is also associated with age of symptom onset in AD, level of brain amyloid burden (a hallmark pathology of AD) [7, 8], and age at onset of amyloid accumulation [9, 10], which precedes clinical symptoms of AD by 10–15 years [11]. In each instance, the *APOE*ε4 allele has been linked to more advanced or more severe disease. Importantly the effect of *APOE* genotype varies considerably across different ethnic groups, with the *APOE*ε4 allele conferring the most risk for individuals of European and East Asian ancestry, compared to, for example, individuals of African ancestry for whom an *APOE*ε4 allele confers less (but still elevated) risk for AD [12].

Despite the clear association between *APOE*ε4 and AD risk, *APOE*ε4 remains a risk allele and is not sufficient for the development of AD, irrespective of one’s genetic ancestry. Studying individuals who do not develop AD despite an *APOEε4* risk allele can reveal critical factors that limit the development of clinical symptoms of AD. The Women’s Health Initiative Memory Study (WHIMS), an ancillary study to the Women’s Health Initiative (WHI) randomized clinical trials of postmenopausal hormone therapy (HT), provides a large, well-characterized sample of women that may be used to examine factors that promote the maintenance of cognitive health, i.e. cognitive resilience, even in the presence of the *APOE*ε4 risk allele. With an average follow-up of 14 years, the WHIMS sample included both women who have demonstrated cognitive resilience beyond the age of 80 years, as well as women who developed cognitive impairment (i.e., non-resilient). In a previous WHI study, we examined 557 *APOE*ε4 women to determine whether unique demographic, health and lifestyle differentiated women who maintained cognitive health despite carrying the ε4 risk allele [13]. Although we found that better general health was a predictor of cognitive resilience, the specific biological pathways that contributed to preserved cognition among older *APOE*ε4 carriers remain elusive [13–15].

In the current study, we extend this approach to investigate whether there is a unique biological profile that characterizes *APOE*ε4 “escapees” who survive to age greater than 80 and maintain cognitive health (cognitive resilience) using a sample of White women enrolled in the WHIMS. Using plasma and serum samples collected at WHI baseline (1996–1999), we applied a targeted biomarker and large-scale proteomic approach to understand the peripheral biological drivers that underly cognitive resilience despite the presence of one or more *APOE*ε4 risk alleles. In particular, we conducted a targeted assessment of markers of AD pathology (amyloid-β_40_ [Aβ_40_] and Aβ_42_) and neuronal injury (neurofilament-light [NfL], total-tau [t-tau]), and applied the Olink proteomic platform to identify the secreted factors in circulation that are associated with cognitive resilience in *APOE*ε4 carriers. We then determined whether the proteomic markers that distinguished *APOE*ε4 cognitively resilient versus cognitively non-resilient women were distinct from those that discriminated *APOE*ε3/3 cognitively resilient women from those who developed cognitive impairment by age 80 (i.e., non-resilient). After identifying candidate proteins in our discovery analyses, we replicated our results in the UK Biobank cohort. Additionally, we performed pathway analyses and conducted a tissue- and cell-specific characterization to interrogate the biology of proteins linked to resilience among *APOE*ε4 carriers.

## Methods

### Participants

This case-cohort study investigated WHIMS participants, who were WHI participants enrolled in the two randomized clinical trials of hormone therapy and were aged 65 and older at enrollment between 1996 and 1999 [16–18]. WHIMS participants were all cognitively unimpaired at baseline. Cognitive status was determined annually through cognitive evaluations at in-person clinic visits through 2008 and subsequently by telephone cognitive assessments through the final cognitive follow-up in 2021. The procedures for diagnostic adjudication of cognitive status have been detailed previously, with diagnoses of probable dementia and mild cognitive impairment comprising the groups with any cognitive impairment [19]. Eligible women for this study were WHIMS participants who had one of the following genotypes: *ε4/ ε4*,* ε4/ε3*,* or ε3*/*ε3* genotype. *APOE* genotype was defined based on the rs429358 and rs7412 SNPs, which were imputed based on the 1000 Genomes Project reference panel and MaCH algorithms implemented in Minimac [13, 20]. Both SNPs had high imputation quality (R2 > 0.97 for rs429358 and R2 > 0.97 for rs7412).

The sampling strategy was based on *APOE* genotype and cognitive status as of April 11, 2019. The sample included all 876 women with cognitive impairment (mild cognitive impairment or probable dementia) and all women with an *APOE*ε4/ε4 (*N* = 54) or *APOE*ε4/*ε3* (*N* = 624) genotype. We also randomly selected 405 women for inclusion, balanced to the unimpaired *APOE*ε4/* women with respect to age at enrollment, from the 1219 cognitively unimpaired women, age > 80 with *APOEε3*/*ε3* genotype. Sixty women with ε2/ε4 alleles were excluded due to opposing effects of *ε2* and ε4 alleles on amyloid accumulation and longevity. The sample was limited to non-Hispanic White women due to different *APOE* genotype frequencies and reduced penetrance of *APOE*ε4 on AD risk in in African-Americans [21]. White race was determined based on self-report. The following 6 groups were selected based on *APOE* genotype (ε4/* vs. *ε3*/*ε3*), age (≤ 80 vs. > 80 at impairment or last follow-up if unimpaired), and cognitive impairment status (unimpaired, cognitively impaired):

- *APOE*ε4/*, age ≤ 80 and cognitively impaired (non-resilient).
- *APOE*ε4/*, age > 80 and cognitively unimpaired (resilient).
- *APOE*ε4/*, age > 80 and cognitively impaired (80 + impaired).
- *APOE*ε3/ε3, age ≤ 80 and cognitively impaired (non-resilient).
- *APOE*ε3/ε3 age > 80 and cognitively unimpaired (resilient).
- *APOE*ε3/ε3 age > 80 and cognitively impaired (80 + impaired).

**Fig. 1:**
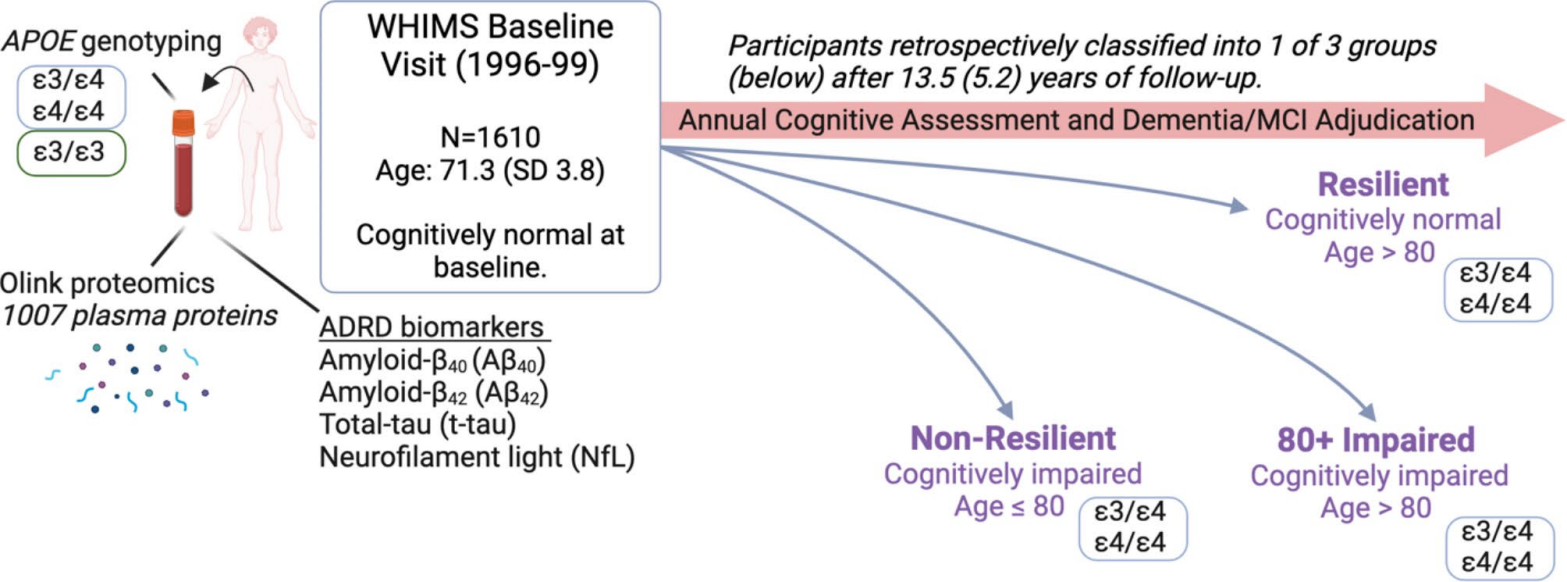
Study design. All participants were cognitively normal at baseline. Targeted ADRD biomarkers (Quanterix) and plasma proteomic assays (Olink) were applied to plasma collected during each participant’s baseline visit. The primary analyses compared resilient to non-resilient *APOE*e4 carriers on targeted Alzheimer’s disease and related dementia plasma biomarkers and plasma proteomic measurements. Figure made with BioRender.com. *Abbreviations.* ADRD, Alzheimer’s disease and related dementias; WHIMS, Women’s Health Initiative Memory Study

The primary analyses compared targeted plasma AD and related dementia (ADRD) biomarkers and plasma proteomic measurements among resilient and non-resilient *APOE*ε4 carriers. These comparisons were repeated for *APOE*ε3 homozygotes to determine whether findings were modified by *APOE* genotype. The study design is illustrated in Fig. 1. Sample characteristics are shown in Table 1 and Supplementary Table 1. Written informed consents were obtained from all participants. The study design, data collection and analyses in this study were approved by the institutional review boards of WHIMS participating centers.

**Table 1:** Demographic and clinical characteristics of *APOE*e4/* participants

	Whole Sample	Non-resilient	Resilient	80 + Impaired
Group description		Age ≤ 80 and cog. impaired	Age > 80 without cog. impairment	Age > 80 and cognitive impairment
N	1610	114	342	214
Age baseline, mean (SD)	71.3 (3.8)	70.7 (3.4)	70.5 (3.6)	72.5 (3.9)
*Education*,* N (%)*				
< High school	80 (5.0)	7 (6.1)	15 (4.4)	9 (4.2)
Highschool/GED	336 (20.9)	34 (29.8)	62 (18.1)	50 (23.4)
Some college	631 (39.3)	41 (36.0)	141 (41.4)	84 (39.3)
College grad	559 (34.8)	32 (28.1)	123 (36.1)	71 (33.2)
Time to dx. or last assessment in years	13.5 (5.2)	5.5 (3.6)	15.5 (3.8)	12.8 (4.4)
*Prior HT use*,* N (%)*				
Never used (0)	849 (52.8)	70 (61.4)	183 (53.5)	101 (47.2)
Past user (1)	644 (40.0)	37 (32.5)	134 (39.2)	96 (44.9)
Current user (2)	116 (7.2)	7 (6.1)	25 (7.3)	17 (7.9)
HT randomization (HT group), N (%)	823 (51.1)	61 (53.5)	176 (51.5)	104 (48.6)
Prior Hysterectomy, N (%)	592 (36.8)	44 (38.6)	121 (35.4)	86 (40.2)
*Clinical characteristics*				
BMI, mean (SD)	28.19 (5.42)	27.82 (5.75)	28.41 (5.93)	27.10 (4.94)
Hypertension, N (%)	769 (47.8)	67 (58.8)	164 (48.0)	94 (43.9)
Diabetes, N (%)	105 (6.5)	12 (10.5)	17 (5.0)	8 (3.7)
High cholesterol, N (%)	301 (19.0)	24 (21.2)	75 (22.2)	37 (17.5)
Stroke, N (%)	17 (1.06)	4 (3.51)	2 (0.58)	1 (0.47)
eGFR CKD-EPI, mean (SD)	80.0 (12.7)	81.1 (14.8)	80.3 (12.5)	79.8 (12.0)
Baseline 3MS, mean (SD)	95.5 (4.2)	92.3 (5.9)	96.5 (3.2)	95.4 (4.0)
Incident MCI, N (%)	489 (30.4)	65 (57.0)	0 (0)	107 (50%)
Incident dementia, N (%)	374 (23.2)	49 (43.0)	0 (0)	107 (50%)

Four participants missing education, 1 missing prior hormone therapy use, 1 missing hormone therapy randomization, 7 missing BMI, 22 missing high cholesterol, 12 missing eGFR, 17 missing baseline 3MS

*Abbreviations*: 3MS, modified mini-mental state test; BMI, body mass index; CKD-EPI, chronic kidney disease epidemiology collaboration; eGFR, estimated glomerular filtration rate; HT, hormone therapy; SD, standard deviation

### Cognitive assessment

Cognitive status was determined by central adjudication by a panel of experts that included a neurologist, geriatric psychiatrist, and geropsychologist, using a screening procedure that triggered a detailed neuropsychological evaluation and proxy reports of functioning. Participants were classified as having probable dementia, MCI or no impairment (Cognitively Unimpaired; CU) [22, 23]. As noted above, individuals with probable dementia or MCI were included in the cognitively impaired group.

### Blood sampling and biomarker measurement

Blood samples were collected at WHI baseline (1996–1999), and plasma and serum samples were stored using standardized protocols, as described elsewhere (www.whi.org). We used the high sensitivity Quanterix single molecule array (Simoa) platform to quantify plasma and serum markers of AD pathology (Aβ_40_, Aβ_42_), neuronal injury (NfL, t-tau), and inflammation (interleukin 6 [IL-6]). Plasma Aβ_40_, Aβ_42_, and t-tau were quantified using the N3PA assay (Quanterix). Serum NfL was quantified using the singleplex NfL assay (Quanterix). Serum IL6 was quantified using the singleplex IL-6 assay (Quanterix). All Quanterix assays were performed in duplicate on the HD-X platform, and values were averaged. IL-6 was natural log transformed due to a skewed distribution. Outliers, defined as > 5 SD from the sample mean, were winsorized. The mean intra-assay CVs were 2%, 2%, 8%, 4%, and 6% for Aβ_40_, Aβ_42_, t-tau, NfL, and IL-6, respectively. The mean inter-assay CVs were 10%, 12%, 20%, 11%, and 17% for Aβ_40_, Aβ_42_, t-tau, NfL, and IL-6.

Plasma proteomic profiling was conducted using the Olink multiplex proximity extension assay (PEA) panels according to the manufacturer’s instructions and as previously described [24] at Olink Proteomics (Olink Bioscience, Sweden; Watertown, MA). The abundance of 1104 analytes (1069 unique proteins) was assessed by PEA using 12 Olink panels: Cardiometabolic, Cardiovascular II, Cardiovascular III, Cell Regulation, Development, Immune Response, Inflammation, Metabolism, Neuroexploratory, Neurology, Oncology II and Organ Damage. All samples were randomized and intensity normalized with a single lot used for each Target panel across the entire study. PEA is a dual-recognition immunoassay, where two matched antibodies labelled with unique DNA oligonucleotides simultaneously bind to a target protein in solution [24]. The corresponding oligonucleotides form an amplicon allowing for quantification of protein expression by microfluidic qPCR using Fluidigm BioMark HD system (Fluidigm Corporation, South San Francisco, California). Four internal controls are added to each sample to monitor the quality of assay performance, as well as the quality of individual samples. Each sample plate is evaluated using the standard deviation of the internal controls (below 0.2 on normalized protein expression (NPX)). The quality of each sample is assessed by evaluating the deviation from the median value of the controls for each individual sample. Samples that deviate less than 0.3 NPX from the median pass the quality control. Data are presented as normalized protein expression values, Olink Proteomics’ arbitrary unit on a log2 scale adjusted for inter-plate variations of internal controls within each sample and external controls between samples.

Protein measurements that did not meet Olink quality control standards were excluded. Of the 1104 Olink-measured proteins, 97 were excluded from all analyses due to the large proportion (> 30%) of missing measurements. Protein measurements were considered missing when the protein measurement (i) was below the lower limit of detection (LOD) or (ii) did not meet quality control standards. The percentage of missing proteins did not differ meaningfully between groups (Supplementary Table 2). For proteins that had measurements below the lower LOD with ≤ 30% of proteins measurements missing, values below LOD were imputed using a value of LOD/2 [25]. Outliers, defined as > 5 standard deviations from the sample mean, were winsorized. A total of 1007 proteins were included in this analysis. The median intraclass correlation (ICC) calculated using 82 blind duplicates was 0.90 for all proteins (Q1 = 0.82, Q3 = 0.95). While the proteins on the Cardiometabolic panel had a median ICC of 0.73, proteins on each of the other 11 panels had a median ICC of approximately 0.90 (Supplementary Fig. 1). Olink quality control details are provided in the Supplementary Methods.

Orthogonal validation of proteomic measurement relied on cis-protein quantitative trait loci (cis-pQTLs) [26, 27]. Specifically, cis-pQTLs have been identified at genome-wide significance for 19 of the 20 proteins associated with resilient vs. non-resilient status among *APOE*ε4 carriers, 14 of which are sentinel cis-pQTLs. Similarly, genome-wide significant cis-pQTLs have been identified for 11 of the 12 proteins associated with resilient vs. non-resilient status in *APOE*ε3 carriers, 8 of which are sentinel cis-pQTLs (Supplementary Table 3) [26, 27].

### Covariates

Primary analyses adjusted for self-reported age at enrollment and years of education, recruitment region, WHI hormone therapy arm (HT; treatment/placebo), history of hypertension or diabetes, obesity (body mass index ≥ 30), high cholesterol (> 200 mg/dL), and estimated glomerular filtration rate (eGFR)-creatinine. eGFR was calculated based on serum creatinine, age, sex, and race, as defined by the CKD-EPI equation [28]. Hypertension, diabetes, obesity, and high cholesterol were assessed using standard protocols. Race and sex were not included as covariates because all participants in the current analyses were white and female.

### External replication

We used data from the UK Biobank Study to replicate the results of our discovery analyses. As part of the UK Biobank Plasma Proteomics Project (UKB-PPP), proteins were measured in plasma for 54,219 participants using the Olink Explore 3072 platform. After extensive quality control, technical and biological validations, data on 2,923 assays were made available. Blood samples used for plasma proteomics were collected at the study entry between 2006 and 2010. Dementia diagnoses were ascertained primarily from hospital inpatient records (Hospital Episode Statistics [HES] data from England, Scotland, and Wales; censored to 31 October 2022, 31 August 2022, and 31 May 2022, respectively) [29]. Non-white participants, which made up approximately 1% of the sample, were excluded from the UK Biobank analyses, consistent with the discovery analyses. A total of 35,494 participants who attained the age of 65 (or were diagnosed with dementia) by the date of censoring were included in this analysis. Please see the Supplementary Methods for details.

### Statistical analysis

Two-way ANCOVA models with each biomarker as the outcome were used to compare non-resilient and 80 + impaired participant groups with resilient participants by *APOE*ε4 status on measures of targeted AD and neurodegeneration biomarkers. The main predictors included *APOE*ε4 status (*APOE*ε4/* vs. *APOE*ε3/ε3), cognitive status (resilient vs. non-resilient vs. 80 + impaired), and *APOE*ε4 status by cognitive status interaction. Covariates included baseline age, recruitment region, HT treatment (active vs. placebo), education, obesity, kidney function (eGFR), diabetes, and high cholesterol. This model setup allowed us to estimate and test group differences in biomarker level by cognitive status among *APOE*ε4 carriers, or the interaction between *APOE*ε4 status by cognitive status using linear combinations of the regression coefficients from the model after adjusting for covariates. The same model setup was used for proteomic analyses to compare the baseline plasma protein abundance of resilient women to that of non-resilient women among *APOE*ε4 carriers. For proteome-wide analyses, an FDR-corrected *P* < 0.05 was used to establish statistical significance, whereas unadjusted *P* < 0.01 was considered the threshold for suggestive associations. For proteins showing group differences at the suggestive unadjusted *P* < 0.01 threshold, we determined whether *APOE*ε4 status modified the difference in plasma protein abundance between resilient and non-resilient participants. We performed parallel analyses based on proteins that differentiated resilient from non-resilient *APOEε*4 noncarrier women using the same covariates and follow-up analyses to determine the moderating effect of *APOE* genotype. We next examined the age-adjusted Spearman correlation between candidate proteins and ADRD (i.e., Aß_42/40_, tau, NfL, Aß_42/40_/tau) and inflammatory (IL6) biomarkers in the full sample and separately among *APOE*ε4 carriers and *noncarriers* groups. Statistical methods used for functional characterization of candidate proteins are provided in the Supplementary Methods. Analyses were conducted using SAS 9.4 (Cary, NC).

## Results

A total of 1610 WHIMS participants were included in the analytic sample (baseline age: 71.3, [3.8 SD] years; all women; all White); 670 (42%) of these women carried one or more *APOEε*4 alleles. All other included participants (*N* = 940; 58%) were homozygous carriers of the *APOEε*3 allele. Participant demographic characteristics are provided in Table 1 for *APOEε*4 carriers and in Supplementary Table 1 for *APOEε*3 homozygotes. Among *APOEε*4 participants, *N* = 114 were classified as non-resilient (incident cognitive impairment, age ≤ 80), *N* = 342 as resilient (age > 80 without incident cognitive impairment), and *N* = 214 as 80 + impaired (incident cognitive impairment, age > 80). Among *APOEε*3 homozygous participants, *N* = 104 were classified as non-resilient, *N* = 405 as resilient, and *N* = 431 as 80 + impaired.

### Groups differ on targeted ADRD biomarkers

**Fig. 2:**
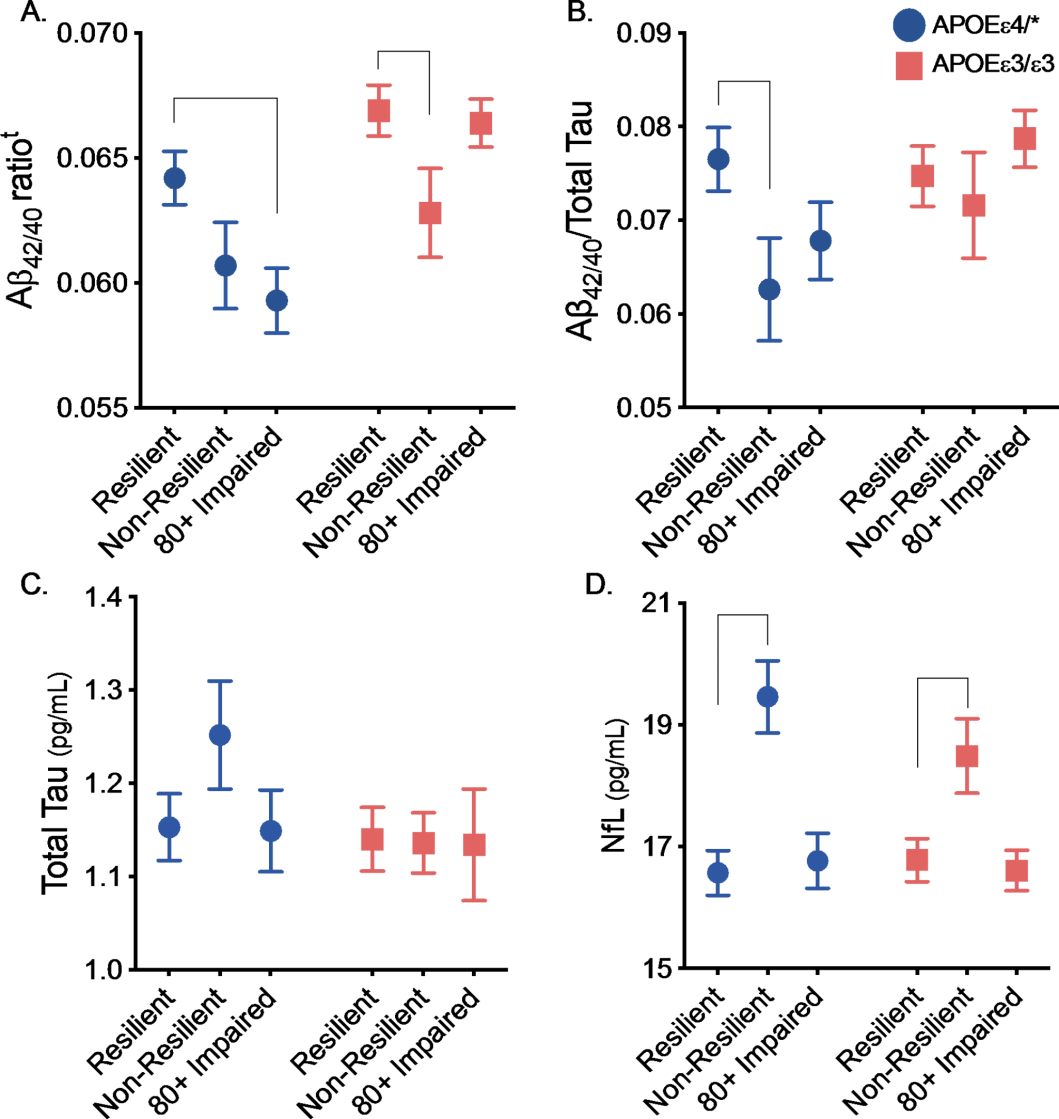
Alzheimer’s disease and neurodegeneration biomarker levels among resilient, non-resilient, and 80 + impaired participants. All results were derived from an 2 × 3 (*APOE* genotype x cognitive status group) ANCOVA adjusted for baseline age, recruitment region, HT treatment (HT vs. Placebo), education, kidney function (eGFR), diabetes, and high cholesterol. Full results of these analyses are presented in the Supplementary Tables. Results derived from post-hoc comparison of the non-resilient and 80 + impaired groups to the resilient (reference) group ^t^ Interaction between *APOE* genotype and resilient versus 80 + imparted was statistically significant for plasma Aß_42/40_ level (interaction-*P* = 0.03). *Difference between group and resilient group significant at *P* < 0.05

We first examined whether the abundance of plasma Aß_42/40_, Aß_42/40_/t-tau, t-tau, and NfL among resilient *APOEε*4 carriers differed from that of non-resilient *APOEε*4 carriers and 80 + impaired *APOEε*4 carriers. Compared to resilient participants, non-resilient participants had significantly lower Aß_42/40_/tau ratio (indicative of greater cortical Aß) and significantly greater NfL at baseline (Fig. 2; Supplementary Table 4). Compared to resilient participants, 80 + impaired showed lower abundance of Aß_42/40_ at baseline; however, this trend was not observed in *APOEε*3 homozygotes (*P*-interaction = 0.03). Among *APOEε*3 homozygotes, only non-resilient participants had lower Aß_42/40_ abundance and greater NfL at baseline (Fig. 2; Supplementary Table 4). Thus, compared to individuals who remain cognitively resilient, those who develop cognitive impairment before age 80 (i.e., non-resilient individuals) have evidence of greater brain amyloid pathology and neuronal injury, even before the onset of cognitive symptoms. This finding holds for *APOE*ε4 carriers and *APOE*ε3 homozygotes.

### Proteomic analysis of resilient vs. non-resilient *APOEε*4 carriers

**Fig. 3:**
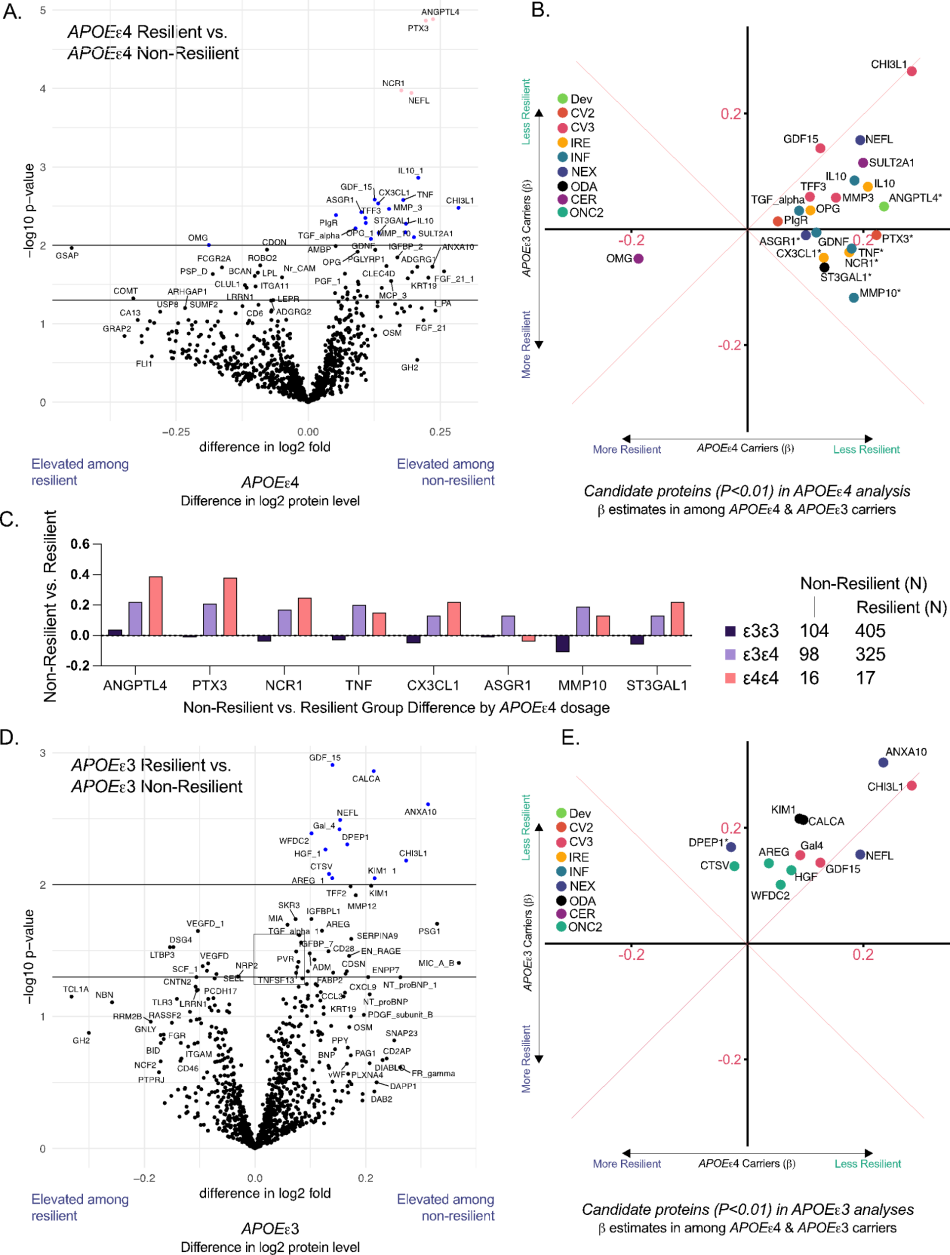
Difference in baseline plasma protein level between resilient and impaired participants. **A**. The adjusted difference between resilient and non-resilient *APOE*ε4 groups. Two horizontal reference lines indicate *P* value of 0.05 and 0.01. **B**. Effect sizes for candidate proteins that differed in our comparison of resilient to non-resilient *APOE*ε4 participants. Effect sizes derived from *APOE*ε4 participants are displayed on the x-axis, whereas effect sizes derived from *APOE*ε3 participants are displayed on the y-axis. * Indicates a statistically significant interaction by *APOE* genotype. **C**. Examination of dose response effects for proteins that were (i) differentially expressed between resilient and non-resilient *APOE*ε4 participants and (ii) showed statistically significant interaction by *APOE* genotype. Dose response was determined by deriving the difference between resilient and non-resilient protein levels among *APOE*ε3/ε3, *APOE*ε3/ε4, and *APOE*ε4/ε4 participants. **D**. The adjusted difference between resilient and non-resilient *APOE*ε3 groups. Two horizontal reference lines indicate p value of 0.05 and 0.01. **E**. Effect sizes for candidate proteins that differed in our comparison of resilient to non-resilient *APOE*ε3 participants. Effect sizes derived from *APOE*ε4 participants are displayed on the x-axis, whereas effect sizes derived from *APOE*ε3 participants are displayed on the y-axis. * Indicates a statistically significant interaction by *APOE*ε4 genotype. All results were derived from an 2 × 3 ANCOVA adjusted for baseline age, recruitment region, HT treatment (HT vs. Placebo), education, kidney function (eGFR), diabetes, and high cholesterol

Using the Olink platform, which provided measurement of 1007 unique proteins, we compared the baseline plasma protein signature of resilient participants to that of non-resilient participants. After adjusting for age, HT treatment arm, education, BMI, diabetes, cholesterol, and eGFR, 20 unique proteins differed between resilient and non-resilient *APOEε*4 carriers at the suggestive uncorrected threshold of *P* < 0.01 (Fig. 3A; Supplementary Table 5). Four of these proteins remained significant after correcting for multiple comparisons using a threshold of FDR-corrected *P* < 0.05. These top *candidate* proteins included Angiopoietin-related protein 4 (ANGPTL4), an endothelial protein involved in glucose and lipid regulation, pentraxin-related protein (PTX3), an innate immune protein involved in regulating complement activation and inflammation, natural cytotoxicity triggering receptor 1 (NCR1), a receptor on natural killer (NK) cells, and neurofilament light (NfL), a major structural component of neurons and marker of neuronal injury. A description of each protein is provided in Supplementary Table 6. Using the same uncorrected *P* < 0.01 threshold, only 3 of the 20 candidate proteins (NfL, GDF15, CHI3L1 [aka YKL40]) also differed between resilient and non-resilient *APOE*ε3 homozygotes. Effect sizes for these three proteins were similar across *APOE*ε4 and *APOE*ε3 homozygotes in terms of magnitude and direction (Fig. 3B; Supplementary Table 5).

We next determined whether *APOE* genotype (*APOEε*4 carriers vs. *APOEε*3 homozygotes) modified the resilient vs. non-resilient differences in plasma protein expression. Eight of the 20 candidate proteins differentially expressed between resilient vs. non-resilient groups showed evidence for effect modification by *APOE* genotype (Fig. 3B; Supplementary Table 5). Proteins affected by *APOE* genotype included the top 3 (ANGPTL4, PTX3, and NCR1), as well as 5 other suggestive proteins (TNF, CX3CL1, ASGR1, MMP10, and ST3GAL1). At least half of the 8 proteins are known to have prominent roles in immune function (Supplementary Table 5). For proteins ANGPTL4, PTX3, NCR1, CX3CL1, and ST3GAL1, we found a stepwise increase in effect size with each additional *APOE ε*4 allele. Although the small sample size precluded formal statistical testing, these results suggest a dose-dependent effect of *APOEε*4 allele possession (Fig. 3C; Supplementary Table 7).

After establishing there is a proteomic signature associated with resilient vs. non-resilient status among *APOEε*4 carriers, we conducted a set of exploratory analyses to determine whether the identified proteins could be used to accurately predict which participants would remain cognitively resilient over the 13.0-year (SD 5.7) follow-up period (see Supplementary Methods). Using a random forest machine learning approach with five-fold cross-validation to classify resilient vs. non-resilient status, we tested four separate models, each with different combinations of prediction features [30]. Models included (a) all proteins significant at a suggestive *P* < 0.01 threshold, (b) all proteins significant at a suggestive *P* < 0.01 threshold and targeted ADRD biomarkers, (c) all proteins significant at a suggestive *P* < 0.01 and age, and (d) all available proteins and targeted ADRD biomarkers. As shown in Supplementary Tables 8 and Supplementary Figs. 2–5, the model with the best classification accuracy included the 20 proteins associated with resilient vs. non-resilient status among *APOE*ε4 carriers at *P* < 0.01 in our discovery analysis and targeted ADRD biomarkers. This model, which included Aß_42/40_ ratio, PTX3, OPG, and ANGPTL4 as the most important parameters, yielded an AUC of 0.66, suggesting poor predictive accuracy.

### Proteomic analysis of resilient vs. non-resilient *APOEε*3 homozygotes

While the previous analysis was based on proteins showing significant differences between *APOEε*4 resilient vs. non-resilient groups, we performed a parallel analysis for *APOEε*3 homozygotes. After covariate adjustment, 12 proteins differed between resilient and non-resilient *APOEε*3 homozygotes at the suggestive threshold of uncorrected *P* < 0.01 (Fig. 3D). The top differentially expressed proteins were Growth differentiation factor 15 (GDF15), a metabolically relevant immune protein, Calcitonin related polypeptide alpha (CALCA), a calcium regulation protein, and Annexin A10 (ANXA10), a protein involved in calcium ion binding. Results for the top 12 proteins are provided in Supplementary Tables 9 and a description of each protein is provided in Supplementary Table 6. Only 1 of the 12 candidate proteins (DPEP1) differentially expressed between resilient vs. non-resilient groups showed evidence for effect modification by *APOE* genotype (Fig. 3E; Supplementary Table 9).

### External replication: candidate proteins and incident dementia

**Fig. 4:**
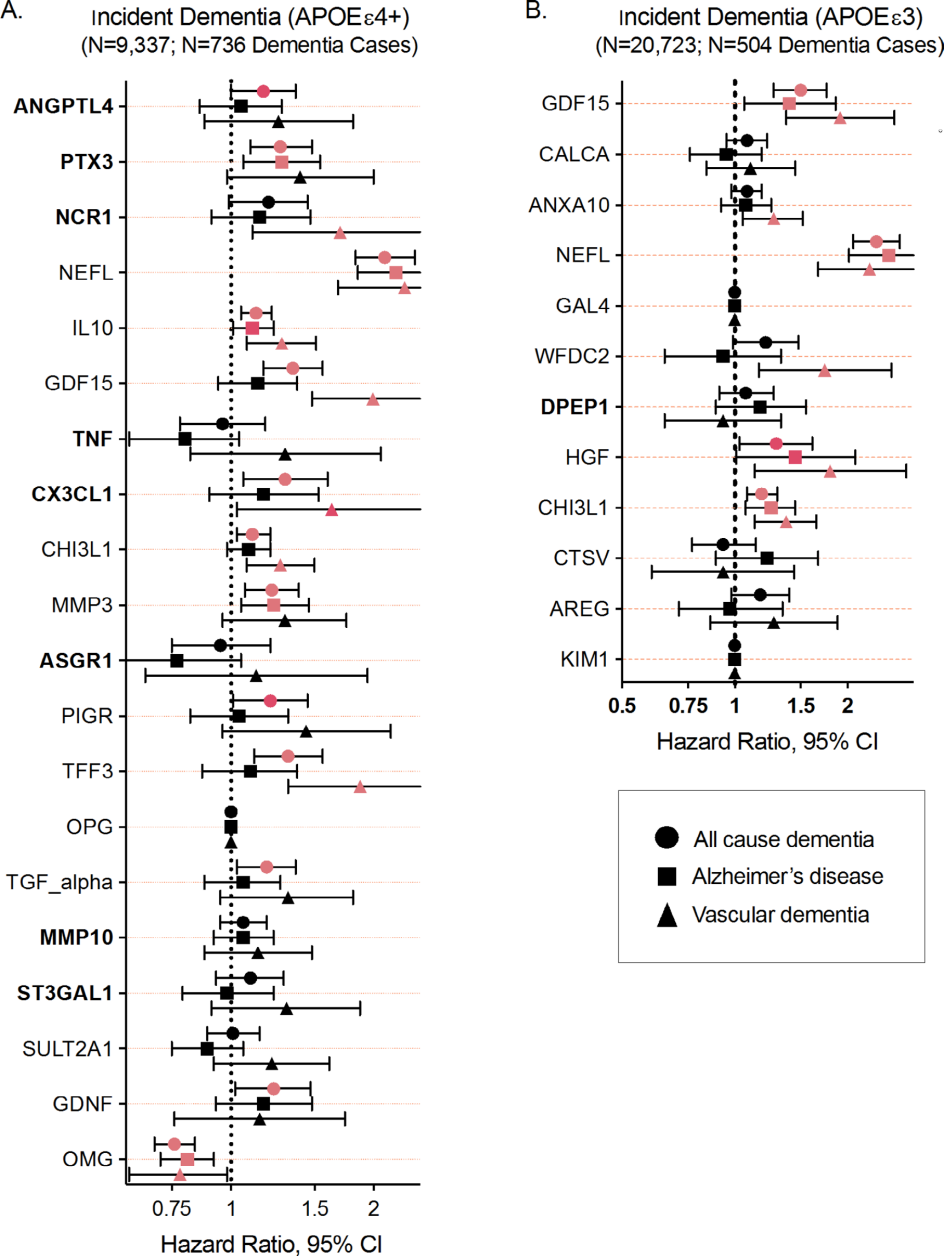
Association of candidate proteins with incident all-cause, Alzheimer’s, and vascular dementia in the UK Biobank. **A**. Association of *APOE*ε4 candidate proteins with incident dementia in the UK Biobank. **B**. Association of *APOE*ε3 candidate proteins with incident dementia in the UK Biobank. Hazard ratios were derived from Cox proportional hazards models adjusted for age, sex, education study site, BMI, kidney function (eGFR), diabetes, and cholesterol. Pink circles represent statistical significance at an unadjusted *P* < 0.05. A bolded protein name indicates significant effect modification by *APOE*ε4 genotype in the WHIMS discovery analyses

We next examined whether proteins associated with resilient vs. non-resilient status in the discovery analysis were also associated with incident all-cause dementia in 35,494 White UK Biobank participants (*N* = 35,985; mean age 60.7 [SD 5.2] at baseline assessment; 54% women; 26% *APOEε*4; Supplementary Table 10). Nineteen of the 20 *APOEε*4 resiliency-associated proteins identified in the discovery analysis were measured in 9,420 non-demented UK Biobank *APOEε*4 carriers using the Olink platform. In analyses adjusting for demographic factors, kidney function, and cardiovascular risk factors, we related each protein to incident dementia over a median follow-up time of 13.8 years (SD 0.8). Thirteen of the 19 (68%) candidate proteins were significantly associated with incident all-cause dementia, including three of the top four risk proteins for *APOEε*4 carriers, ANGPTL4, PTX3, and NfL (Fig. 4A; Supplementary Table 11). Ten of the 12 proteins associated with resiliency among *APOEε*3 homozygotes in our discovery analyses were measured in 25,624 non-demented UK Biobank *APOEε*3 homozygotes. After adjusting for confounders, 4 of 10 candidate proteins (40%) were significantly associated with incident all-cause dementia (Fig. 4B; Supplementary Table 12). All replicated associations in UK Biobank were directionally consistent with the results of the WHIMS discovery analyses.

Secondary analyses examined protein associations with incident AD and VaD, two dementia subtypes defined based on suspected etiology. Heterogeneity in the magnitude of the protein-dementia associations was observed among *APOEε*4 carriers for one NCR1 (one of the top four candidate proteins), GDF15, CHI3L1, and TFF3, each of which showed significant associations with VaD and comparatively weaker associations with AD dementia (Fig. 4A; Supplementary Table 11). A similar pattern was observed for candidate proteins ANXA10 and WFDC2 among *APOEε*3 homozygotes (Fig. 4A; Supplementary Table 12). Ten of the 19 (53%) *APOEε*4 candidate proteins replicated in analyses restricted to *APOEε*4 women participants in the UK Biobank (Supplementary Fig. 6 and Supplementary Tables 13–14). Six of the 19 (32%) associations remained significant when analyses were extended to men (Supplementary Tables 15–16).

### Candidate proteins and targeted ADRD biomarkers

**Fig. 5:**
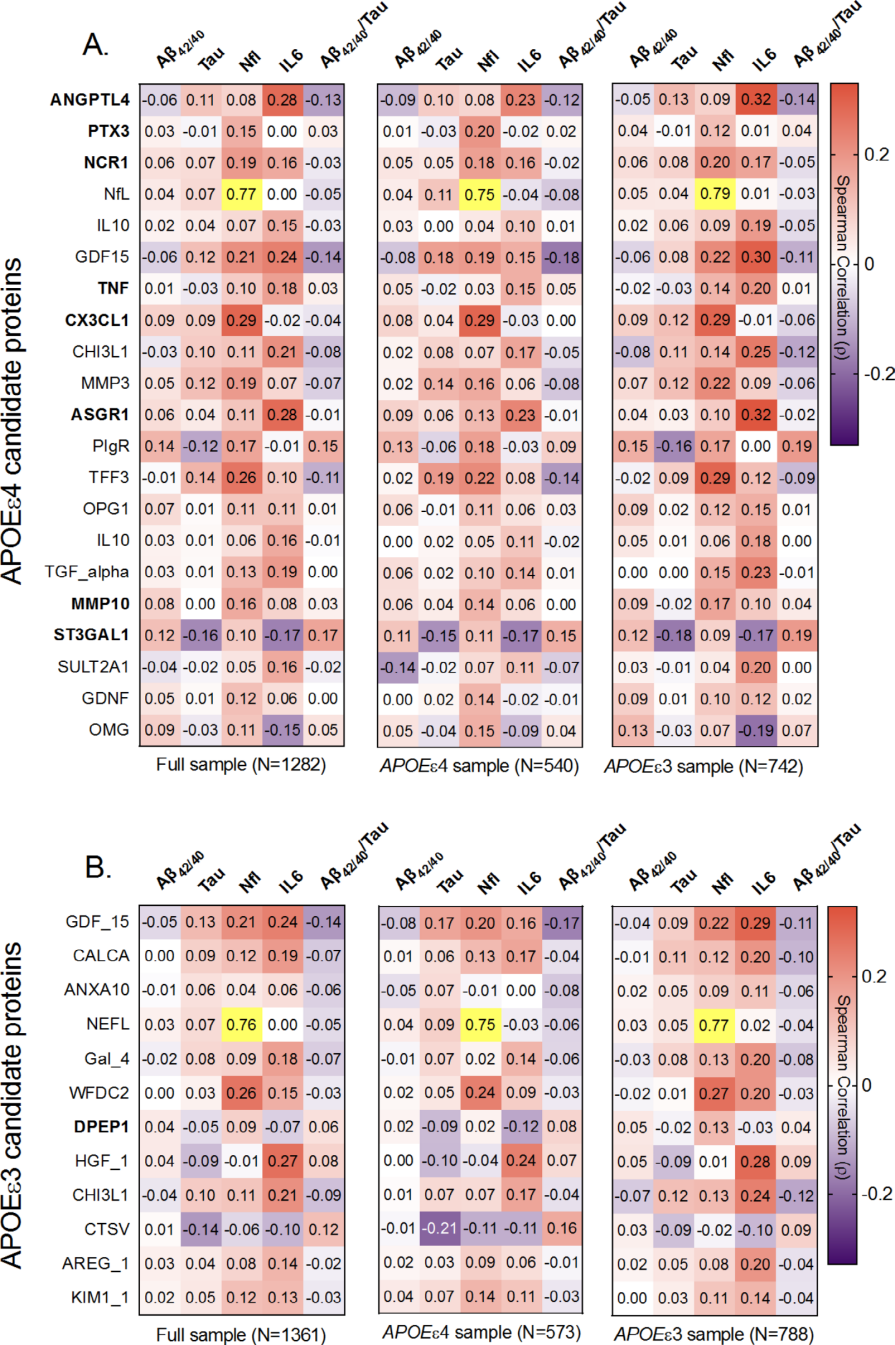
Association of resiliency-associated proteins with plasma ADRD biomarkers **A**. Age-adjusted correlation between plasma protein level and plasma Aß_42/40_, tau, NfL, IL6 and Aß_42/42_ to tau ratio for *APOE*e4 candidate proteins. **B**. Age-adjusted correlation between plasma protein level and plasma Aß_42/40_, tau NfL, IL6 and Aß_42/42_ to tau ratio for *APOE*e3 candidate proteins. The heatmaps display the results in the full sample, among *APOE*e4 carriers, and among *APOE*e3 carriers. Bolded protein name indicates significant effect modification by *APOE* genotype in the discovery (WHIMS) analyses

We next examined whether proteins identified as differentially expressed in our comparison of resilient vs. non-resilient *APOE* ε4 carriers correlated with targeted plasma ADRD biomarkers (Aß_42/40_, t-tau, NfL, and Aß_42/40_/tau) and IL-6, an inflammatory biomarker, after adjusting for age (Fig. 5A-C; Supplementary Table 17). Protein ANGPTL4, which most strongly differentiated resilient from non-resilient *APOEε*4 carriers, was most strongly associated with IL-6 (*r* = 0.28; *P* < 0.0001) in the full sample, supporting the link between ANGPTL4 and inflammation. Notably, ANGPTL4 showed similar correlations with IL6 among *APOEε*4 (*r* = 0.23; *P* < 0.0001) and *APOE*ε3 homozygous women (*r* = 0.32; *P* < 0.0001). The other top candidate protein, PTX3, correlated only with NfL (*r* = 0.15; *P* < 0.0001), whereas NCR1 correlated with both NfL (*r* = 0.19; *P* < 0.0001) and IL-6 (*r* = 0.16; *P* < 0.0001). Both PTX3 and NCR1 proteins correlated similarly with targeted biomarkers across *APOE* genotype. The protein GDF15, which showed the greatest degree of differential expression in the comparison of resilient vs. non-resilient *APOEε*3 homozygotes was most strongly correlated with IL-6 (*r* = 0.24; *P* < 0.0001) and NfL (*r* = 21; *P* < 0.0001) in the full sample (Fig. 5A). Notably, the association of GDF15 with IL-6 was nearly twice as strong among *APOEε*3 homozygotes (*r* = 0.29; *P* < 0.0001) as it was among *APOEε*4 carriers (*r* = 0.16; *P* < 0.0001; Fig. 5B-C; Supplementary Table 18), suggesting that genetic predisposition for AD may influence the responsiveness of GDF15 – a stress response protein with known immunosuppressive function – to IL6-mediated inflammation.

### Functional characterization of *APOEε*4 resiliency-associated proteins

**Fig. 6:**
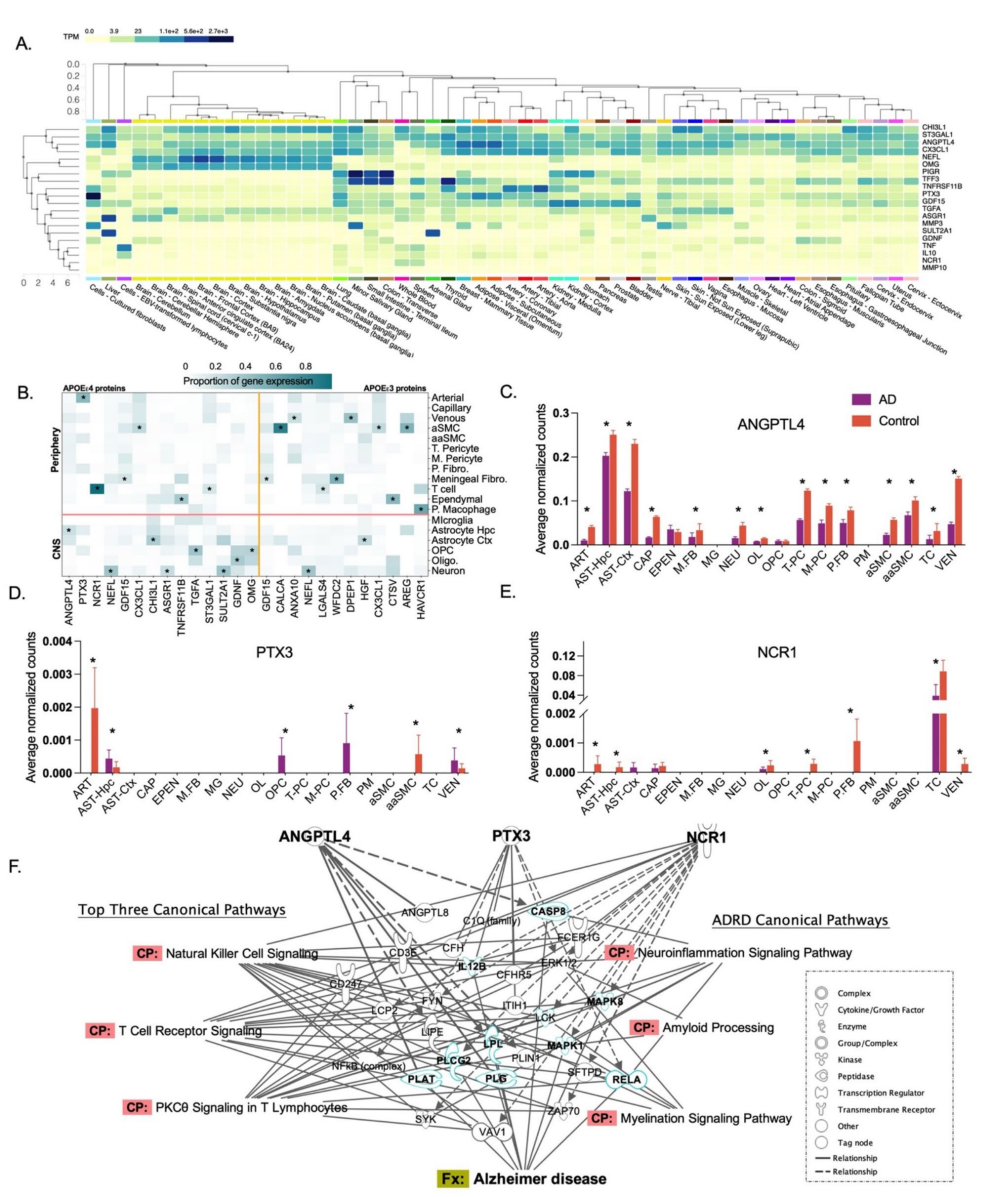
Functional characterization of *APOEε*4 resiliency-associated proteins. **A.** GTEx database gene expression in brain, whole blood, and other tissues using data available from postmortem samples [57]. The expression of cognate genes coding for proteins associated with non-resilient versus resilient status are displayed in transcripts per million. Genes and tissues are grouped on the y- and x-axis based using hierarchical clustering. **B**. Brain and vascular cell-specific expression of genes coding for proteins associated with non-resilient versus resilient status [58]. Values are expressed in terms of average normalized counts. https://twc-stanford.shinyapps.io/human_bbb/. **C**-**E**. Brain and vascular cell-specific expression of genes coding for proteins associated with non-resilient versus resilient status. Expression values are presented stratified by Alzheimer’s disease verses control status [58]. **F.** Using Ingenuity Pathway Analysis (IPA), we identified molecules downstream of top candidate proteins associated with resilient versus non-resilient status among *APOE*e4 carriers. Relationships between molecules (edges) were defined based on activation, causation, chemical-chemical interactions, chemical-protein interactions, inhibition, modification, molecular cleavage, phosphorylation, protein-DNA interactions, protein-protein interactions, protein-RNA interactions, regulation of binding, RNA-RNA interactions, transcription, translocation, and ubiquitination. We identified the top three canonical pathways (based on number of overlapping molecules) among the candidate proteins and downstream molecules (left) and included three canonical pathways implicated in Alzheimer’s disease and related dementia (right). Bolded molecules are downstream of one or more candidate protein and implicated in AD. *Abbreviations*: aaSMC, Arteriolar Smooth Muscle Cell; ART, Arterial; aSMC, Vascular Smooth Muscle Cell; AST-Ctx, Astrocyte-Cortex; AST-Hpc, Astrocyte-hippocampus; CAP, Capillary; EPEN, Ependymal; M.FB, Meningeal Fibroblast; MCR, Motoric Cognitive Risk; MG, Microglia; M-PC, ECM-regulating Pericyte; NEU, Neuron; OL, Oligodendrocyte; OPC, Oligodendrocyte Precursor Cell; P.FB, Perivascular Fibroblast; PM, Perivascular Macrophage; TC, T-cell; T-PC, Solute transport-Pericyte; VEN, Venous; VINE, Vessel Isolation and Nuclei Extraction for Sequencing

We used available data from the GTEX database to characterize the tissue expression of candidate protein coding genes (Fig. 6A). While many of the candidate protein coding genes are expressed in brain tissue, only two genes – *NEFL* and *OMG* – showed enriched (preferential) gene expression in brain tissue. Cognate gene for the top *APOEε*4 candidate proteins ANGPTL4 and PTX3 were enhanced in adipose tissue, whereas *NCR1* expression was enriched in lymphoid tissues. Examination of publicly available gene expression data for brain, vascular, meningeal, and immune cells revealed that *ANGPTL4* had highest cell-specific expression in astrocytes, whereas *PTX3* and *NCR1* were most highly expression in the arterial cells and T lymphocytes, respectively (Fig. 6B). In our examination of brain, vascular, meningeal, and immune cell types, 8 of 14 (57%) genes coding for *APOEε*4 candidate proteins were most highly expressed in CNS cell types (e.g., neuronal, astrocytic), whereas only 2 of 12 (17%) of genes coding for *APOEε*3 resiliency-associated candidate proteins showed the highest expression in CNS cell types (Fig. 6B; Supplementary Table 19). Among individuals with a pathological AD diagnosis, *ANGPTL4* expression was significantly downregulated in astrocytes, neurons, T cells and other neurovascular cell types (Fig. 6C). PTX3 expression was strongly downregulated in arterial and arteriolar smooth muscle cells and upregulated in oligodendrocyte precursor cells and perivascular fibroblast (Fig. 6D). Prominent reduction of NCR1 expression was found among T lymphocytes in the context of AD pathology (Fig. 6E; Supplementary Table 20).

We identified 25 molecules downstream of top candidate proteins ANGPTL4, PTX3, and NCR1 using Ingenuity Pathway Analyses (Fig. 6F). NK cell, T cell receptor, and PKC- θ signaling pathways were most enriched (based on number of overlapping genes/proteins) among this set of downstream molecules. In total, nine of these downstream molecules have been previously associated with AD, including CASP8, PLG, LPL, PLAT (downstream of ANGPTL4), IL12B, RELA (downstream of PTX3), MAPK1, MAPK8, and PLCG2 (downstream of NCR1; Fig. 6F).

Additionally, we examined the protein-protein interaction networks for the 20 *APOEε*4 and separate for the 12 *APOEε*3 candidate proteins (Supplementary Fig. 7). The largest network, which included 13 *APOEε*4 resiliency-associated candidate proteins – including ANGPTL4, PTX3, and NCR1 – was enriched for chronic inflammation. Notably, 6 (46%) of the proteins in this chronic inflammation cluster were associated with resilient vs. non-resilient status in an *APOE*-dependent manner. This was the case for only two of seven (29%) proteins outside of this cluster.

### Genetic regulation of *APOEε*4 resiliency-associated proteins

Cis- and trans-protein quantitative trait loci (pQTLs) have been identified for ANGPTL4, PTX3, and NCR1 (Supplementary Table 3) [27]. Using the Online Neurodegenerative Trait Integrative Multi-Omics Explorer (ONTIME) [31], we found that the cis-pQTL variant associated with plasma ANGPTL4 abundance (rs2278236) also regulates ANGPTL4 in the cerebral spinal fluid (CSF) (Supplementary Fig. 8). GWAS have identified this same locus as a regulator of blood HDL levels [32, 33]. Together, these findings suggest a genetic coregulation of ANGPTL4 in blood and in CSF in a manner that may influence lipoprotein processing.

## Discussion

Using data from the WHIMS cohort, the current study identified a set of proteins in plasma linked to cognitive resilience among older women carrying one or more copies of the major AD risk variant, *APOE*ε4. Compared to *APOEε*4 carriers who reached age 80 without cognitive impairment (the resilient group), *APOEε*4 carriers who developed cognitive impairment before age 80 (the non-resilient group) showed blood biomarker evidence of greater amyloid burden years before the onset of cognitive impairment. Blood biomarker evidence of greater neurodegeneration (NfL) was also observed among non-resilient participants, but not those impaired at 80+, consistent with the notion that NfL becomes elevated close to the time of symptom onset [34, 35]. These findings were not unique to *APOEε*4 carriers, as non-resilient *APOEε*3 homozygotes also showed greater amyloid burden and neurodegeneration than their resilient counterparts. Compared to resilient *APOEε*4 carriers, non-resilient *APOEε*4 carriers also showed greater abundance of multiple proteins less well characterized in the AD/dementia literature, including a regulator of angiogenesis, glucose homeostasis, and lipid metabolism (ANGPTL4), an activator of complement signaling that is induced by pro-inflammatory cytokines (PTX3), and a natural killer (NK) cell receptor involved in the viral cellular defense response (NCR1). Notably, the associations of ANGPTL4 and PTX3 with incident all-cause dementia, the association of PTX3 with AD dementia, and the association of NCR1 with incident VaD were also observed among *APOEε*4 carriers in the UK Biobank replication cohort. Importantly, our discovery analysis demonstrated that these findings did not extend to *APOEε*3 homozygotes, suggesting that the biology linking these proteins to cognitive impairment may be influenced by *APOE* genotype. While the top three candidate proteins (ANGPTL4, PTX3, and NCR1) showed little to no associations with targeted AD-specific biomarkers (Aß_42/40_ ratio, Aß_42/40_/t-tau ratio), each correlated positively with measures of neuronal injury (NfL) or inflammation (IL6), suggesting that the link between these proteins and cognitive impairment is unlikely to be mediated through known amyloidogenic pathways. This hypothesis is supported by the tendency for candidate proteins to be more strongly associated with incident VaD than incident AD dementia.

The *APOEε*4 allele, the strongest known genetic risk factor for sporadic AD, is associated with a set of unique clinical and pathological traits, including earlier age of dementia onset [3, 36], earlier amyloid deposition [37, 38], greater overall burden of cortical amyloid [39, 40] and cerebral amyloid angiopathy (CAA) [41]. Despite the increased dementia risk conferred by *APOEε*4 allele possession, a sizable proportion of *APOEε*4-positive individuals maintain normal cognitive function into older adulthood [13]. Here, we identified 20 unique proteins that were associated with resilient status at a suggestive (uncorrected *P* < 0.01) threshold. In addition to demonstrating that widely recognized ADRD biomarkers, such as CHI3L1 (YKL-40) [42], NfL [43], and GDF15 [44] are similarly associated with future cognitive impairment among *APOEε*4 carriers and *APOEε*3 homozygotes, we identified a set of 8 proteins to be uniquely predictive of future cognitive impairment among *APOEε*4 carriers. Like ANGPTL4, PTX3, and NCR1, plasma abundance of TNF, CX3CL1, MMP10, ST3GAL1, and ASGR were associated with resilient vs. non-resilient status only among *APOEε*4 carriers. This set of proteins, all of which were upregulated in the at-risk group, may represent a set of biological processes and molecular functions that ultimately determine the extent to which cognition can be preserved in the face of enhanced *APOE-*determined risk for AD and, as suggested by our results, VaD.

Plasma ANGPTL4 most strongly discriminated resilient from non-resilient *APOEε*4 carriers in our discovery analysis and replicated in our analyses of 14-year dementia risk. The *APOE*ε4-specific association of ANGPTL4 with future cognitive impairment may be driven by the joint role of ANGPTL4 and *APOE* in lipid metabolism. ANGPTL4 has been shown to inhibit lipoprotein lipase (LPL) and regulate macrophage and myocyte LPL uptake [45]. Similarly, apolipoprotein E regulates the delivery of cholesterol to cells (including neurons and glial cells) and the clearance of lipoproteins from the blood [45]. The current findings suggest that, among women with *APOE*ε4-associated vulnerabilities in lipid processing, systemic elevation of AGNPTL4 may be consequential for brain health. Inconsistent with the upregulation of ANGPTL4 in plasma among those at risk for cognitive impairment, autopsied AD brains (compared to non-AD control brains) showed reduced ANGPTL4 expression in astrocytes, neurons, cerebrovascular cell types, and meningeal fibroblast. While it is possible that elevations in plasma ANGPTL4 result from an efflux of the ANGPTL4 protein from the CNS, ANGPTL4 is ubiquitously expressed across tissue types, so this cannot be known for certain.

Two immune proteins, PTX3 and NCR1, were also identified as top candidate proteins upregulated in plasma among non-resilient *APOE*ε4 carriers in our discovery analysis. PTX3, a long-form pentraxin, functions as a pattern recognition receptor and regulator of complement activation [46] and vascular inflammation [47]. PTX3 has been previously identified as a major component of the glial secretome in response to LPS and INF-gamma stimulation and as a modulator of microglia phagocytic function [48]. A previous study found plasma PTX3 to be associated with cognitive decline in older women, but not men, suggesting that the association between this protein and brain function may occur through a sex-specific process [49]. The current study extends previous work by demonstrating that PTX3 may be particularly relevant to cognitive function among *APOE*ε4 carriers. NCR1, a receptor expressed on NK cells, has not been well characterized in the context of ADRD. Here, we showed prominent and specific expression of NCR1 in immune cell types, particularly T cells. Further analyses demonstrated that T cell expression of NCR1 was attenuated in patients with AD. NK cells, like T cells, are effector lymphocytes that participate in antiviral cellular immunity. Although peripheral T cell infiltration into the CNS has been identified as a potential driver of AD, particularly tau progression [50], whether peripheral or central NK cell activity, or NCR1 signaling in particular, influences risk for dementia remains an area of ongoing investigation [51, 52]. These results highlight several proteins and, by extension, associated biological pathways that are implicated in developing cognitive impairment in *APOE*ε4 carriers. Each of these proteins and pathways warrant further study to determine their mechanistic significance and utility as therapeutic targets. Of note, a drug targeting NCR1 (SAR443579), is currently in Phase 1/2 for acute myeloid leukemia (NCT05086315). If the causal association between NCR1 and cognitive impairment in *APOE*ε4 carriers can be established, this may represent a drug repurposing opportunity.

While our candidate proteins and protein networks provide insight into the biology underlying cognitive resilience in *APOE*ε4 carriers, we show here that plasma proteins alone – or in combination with Aß_42/40_, total tau, and NfL – do not accurately predict which *APOE*ε4 carriers will remain free of cognitive impairment over the next one to two decades. Etiological heterogeneity, use of a mixed cognitive outcome (MCI or dementia), and the extended follow-up period are factors that may have limited the predictive power of the identified proteins. Our findings suggest there is a sizable amount of variation in cognitive impairment risk that is not captured by this portion of the plasma proteome.

The current study has several strengths including the unique cognitively healthy longevity case-cohort study design, the prospective determination of cognitive status, the use of high throughput proteomic data, and the replication in a large, well-characterized, independent cohort. Nevertheless, our results should be interpreted within the context of several limitations. First, cognitive impairment in our discovery analysis, which included MCI and dementia, was not classified based on etiology. Although we expected AD pathology to be overrepresented among *APOEε*4 carriers, and our targeted analysis of plasma Aß_42/40_ suggests that this is the case, we do not believe we could accurately identify AD pathological change with only plasma Aß_42/40_ [53]. It is possible that some of the effect modification attributed to *APOE*ε4 allele carriage was instead due to varying prevalence of non-AD pathologies. Second, the discovery analysis was restricted to White women because the impact of *APOE*ε4 on AD risk in non-White women is unclear. Replication of a large proportion of candidate proteins using an external cohort helps to address issues of generalizability; yet, whether these results generalize to a more diverse cohort remains unclear, particularly given the varied effects of *APOE*ε4 across persons of distinct ancestries [54]. There is a clear need to validate identified proteins in more diverse populations.

Additionally, there is evidence that socioeconomic status and other social determinants of health may influence one’s resiliency to AD and other neurodegenerative pathologies [55, 56]. Accordingly, future studies should consider social health determinants as potential moderators of links between biological processes and cognitive resiliency. Third, the ability to interpret any protein associations as causal is limited by the observational study design. Accordingly, future efforts are needed to functionally validate the mechanistic role of the candidate proteins and implicate biological pathways with respect to their contribution to risk and resilience among *APOE*ε4 carriers. For example, modulation of peripheral and CNS ANGPTL4, PTX3, and NCR1 levels in human induced pluripotent stem cell-derived neurons or transgenic mice models of amyloidosis (e.g., 5XFAD) or tauopathy (e.g., P301S) with humanized *APOE*ε4 allele would shed additional light on the neurobiological pathways through which these proteins influence AD risk among *APOE*ε4 carriers. Fourth, given the exploratory nature of the machine learning analysis, a formal prediction analysis with feature/predictor selection within the cross-validation procedure was not conducted. Lastly, we note that not all proteins measured on the Olink platform – including several of the identified proteins – have been orthogonally validated. Future studies are needed to establish the consistency of these biomarkers across measurement platforms. Despite the stated limitations, the current study demonstrates that there is a plasma proteomic signature associated with cognitive resiliency among *APOEε*4 carriers, and that this set of proteins is largely unique from the proteins associated with cognitive resiliency among *APOE*ε3 homozygotes.

## Electronic supplementary material

Below is the link to the electronic supplementary material.

Supplementary Material 1

Supplementary Material 2

Supplementary Material 3

## Data Availability

Individual, deidentified participant data from the WHIMS used in these analyses can be obtained by request from the WHI Coordinating Center following a standard application process and approval of a paper proposal and signed data use agreement via the WHI Publications and Presentations Committee. Data will be made available through the WHI online resource, https://www.whi.org/datasets, while the WHI remains funded and indefinitely through BioLINCC, https://biolincc.nhlbi.nih.gov/studies/whi_ctos/.
