# Supplementary material for: Proteomic analysis of APOEε4 carriers implicates lipid metabolism, complement and lymphocyte signaling in cognitive resilience
Source: Mol Neurodegener. 2024 Oct 31;19:81. doi: 10.1186/s13024-024-00772-2 (PMC11526661; doi:10.1186/s13024-024-00772-2)
Supplement: Supplementary file 2 — Supplementary Material 2 [file 13024_2024_772_MOESM2_ESM.pdf]

Supplementary Methods

Title: Proteomic analysis of *APOE*ε4 carriers implicates lipid metabolism, complement and lymphocyte signaling in cognitive resilience

Contents

Olink Quality Control........3
Incubation controls........3
Extension control:........3
Detection control:........3
Inter-plate control:........3
Negative control:........3
Sample Control:........3
Sample QC........3
Plate QC........4
Summary of Quality Control........4
Average %Coefficient of Variation........4
External Replication........5
Study population.........5
Plasma proteomics.........5
APOE genotype.........5
Dementia diagnosis.........5
Statistical analysis.........6
Functional characterization of *APOEε*4 resiliency-associated proteins........6
Gene-tissue expression analysis.........6
Cell-specific expression.........6
Ingenuity Pathway Analysis.........6
Protein-protein interactions.........6
Machine learning methods for prediction of resilient vs. non-resilient status........7
Supplementary Figure 1. Intraclass correlation for proteins on each of the 92-protein Olink panels........8
Supplementary Figure 2. Visualization of SHAP values for prediction of resilient vs. non-resilient status using all proteins significant at a suggestive *P*<0.01 threshold in the previous discovery analysis as model parameters........9
Supplementary Figure 3. Visualization of SHAP values for prediction of resilient vs. non-resilient status using all proteins significant at a suggestive *P*<0.01 threshold in the previous discovery analysis and age at study baseline as model parameters........10
Supplementary Figure 4. Visualization of SHAP values for prediction of resilient vs. non-resilient status using all proteins significant at a suggestive *P*<0.01 threshold in the previous discovery analysis and targeted Alzheimer's disease and related dementia biomarkers ($AB_{42/40}$, total tau, NfL) as model parameters........11
Supplementary Figure 5. Visualization of SHAP values for prediction of resilient vs. non-resilient status using all proteins collected at study baseline as model parameters........12

Supplementary Figure 6. Association of candidate proteins with incident all-cause, Alzheimer's, and vascular dementia among women in the UK Biobank 13

Supplementary Figure 7. Protein interaction network for proteins that significantly differed between resilient and impaired groups among *APOE*ε4 and *APOE*ε3 participants. 14

Supplementary Figure 8. Analysis of protein quantitative trait loci (pQTLs) for ANGPTL4, PTX3, and NCR1 in brain tissues, cerebral spinal fluid, and plasma. 15

Olink Quality Control

The quality control conducted for the Olink® Target 96 biomarker panel is designed to monitor the performance of assays and samples, and account for technical factors that can affect protein measurement. As described in the main text, Olink's QC system used four internal controls that were spiked into every sample and used to monitor three key steps in the Olink protocol: immunoreaction, extension, and amplification/detection [1].

Incubation controls: Incubation Control 1 and Incubation Control 2 are non-human antigens measured using the Proximity Extension Assay (PEA) used to monitor technical variation in each step of the reaction: (i) immunoreaction antibody prob pairs binding to their respective proteins, (ii) extension and pre-amplification, and (iii) the quantification of each biomarker's DNA reporter using high throughput real-time qPCR [1].

Extension control: An antibody coupled to a unique pair of DNA-tags in close proximity so that this control gives a constant signal independent of the immunoreaction. This control is used for extension and amplification monitoring and for adjusting signal from each sample based on the extension and amplification [1].

Detection control: A complete double-stranded DNA amplicon that generates a signal independent of proximity binding or extension. This control is used to monitor the amplification and detection step [1].

In addition, three external controls included in each run.

Inter-plate control: Healthy pooled plasma was included in triplicate on each plate, and the median inter-plate control value was used to normalize each assay and compensate for variation between runs and plates. Inter-plate control plasma included a pool of 92 antibodies, each with a pair of unique DNA-tags positioned in fixed proximity [1].

Negative control: Buffer solution included was in triplicate on each plate. Negative controls were used to identify background noise generated in instances when DNA-tags were in close proximity without previous binding to the appropriate protein. Negative control values were used to set background levels and to determine the limit of detection [1].

Sample Control: Pooled plasma ran in duplicate on each plate. Sample controls are used to calculate the inter- and intra-assay coefficients of variations (CVs), which assess the potential variation between runs and plates, and within plates [1].

Sample QC

The same concentration of each internal control (described above) was spiked into each sample. Therefore, only small deviations in the sample control signal were expected within the same plate. For each sample, the Detection Control and Incubation Control 2 were measured and compared against the median of all samples. Samples with Detection Control and Incubation Control 2 values that differed by more than 0.3 NPX from the sample median were considered QC failures and were excluded from analyses [1].

Plate QC

If the variation across a given plate of any one of three internal controls (Incubation Control 1, Incubation Control 2, and the Detection Control) was considered too large (above 0.2 on normalized protein expression (NPX)), the entire plate was considered unreliable and was excluded from analyses [1].

Summary of Quality Control

Olink Panel Name	No. of samples that passed QC / Tot no. of samples*	Passed samples
CARDIOMETABOLIC	1682 / 1692	99%
CARDIOVASCULAR II	1549 / 1692	92%
CARDIOVASCULAR III	1689 / 1692	100%
CELL REGULATION	1533 / 1692	91%
DEVELOPMENT	1685 / 1692	100%
IMMUNE RESPONSE	1535 / 1692	91%
INFLAMMATION	1556 / 1692	92%
METABOLISM	1684 / 1692	100%
NEURO EXPLORATORY	1554 / 1692	92%
NEUROLOGY	1560 / 1692	92%
ONCOLOGY II	1545 / 1692	91%
ORGAN DAMAGE	1544 / 1692	91%

*Samples displayed here include blind duplicates.

Average %Coefficient of Variation

Olink Panel Name	Intra-Assay %CV (Reference intra CV <15%)	Inter-Assay %CV (Reference Inter CV <25%)
CARDIOMETABOLIC	9	15
CARDIOVASCULAR II	5	15
CARDIOVASCULAR III	8	17
CELL REGULATION	6	12
DEVELOPMENT	6	11
IMMUNE RESPONSE	6	15
INFLAMMATION	7	13
METABOLISM	6	15
NEURO EXPLORATORY	6	11
NEUROLOGY	5	11
ONCOLOGY II	6	12
ORGAN DAMAGE	6	11

External Replication

Study population. We analyzed data from participants from the UK Biobank study, a community-based cohort of 502,387 individuals aged 37 to 73 years at study entry. Blood samples used for plasma proteomics were collected at the study entry between 2006 and 2010. Dementia diagnoses were ascertained primarily from hospital inpatient records (Hospital Episode Statistics [HES] data from England, Scotland and Wales; censored to 31 October 2022, 31 August 2022, and 31 May 2022, respectively), available for all participants, with a subset of participants (45%) having primary care (General Practice [GP]) data, censored between 31 May 2016 to 31 August 2017, depending on data provider (see UK Biobank for information https://biobank.ndph.ox.ac.uk/ukb/exinfo.cgi?src=Data_providers_and_dates). A subset of 52,691 participants had available data on plasma proteomics (as of 15th Feb 2024, the date of analysis). Of these, 52,650 participants were free of dementia at study entry. A total of 48,804 participants had all relevant covariate data. Of these, 35,985 participants who attained the age of 65 (or were diagnosed with dementia) by the date of censoring were included in this analysis. Data used in this study were collected between 2006 and October 2022. The collection and use of UK Biobank data are approved by the Northwest Multi-Center Research Ethics Committee (Research Ethics Committee reference 11/NW/0382). All participants provided informed consent to use their data, health records, and biological materials for research purposes. The present study was conducted under the UK Biobank application number 83534.

Plasma proteomics. We used data from the UK Biobank Plasma Proteomics Project (UKB-PPP) interim release. A complete technical description of the data has been published.[2] In brief, 54,219 participants were selected for inclusion in the UKB-PPP analysis, with protein measured via the Olink Explore 3072 platform. After extensive technical and biological validations, and quality control, data on the first 2,923 assays were made available to researchers in May 2023. Imputation using `impute.knn` from R package {impute} (v1.64.0) was performed to reduce missingness, following the procedure described previously.[3]

APOE genotype. *APOE* ε2 and ε4 genotype were inferred from variants rs7412 and rs429358, respectively. Both were directly genotyped on the Affymetrix Axiom/BiLEVE microarray platform in 488,377 participants.[4]

Dementia diagnosis. Dementia diagnoses were ascertained from HES as ICD-9 or ICD-10 codes in the whole cohort as well as primary care data as Read v2 or CTV3 codes in 45% of the cohort (https://biobank.ndph.ox.ac.uk/ukb/label.cgi?id=3000). Specific diagnostic codes for dementia were identified from the UK NHS National Institute for Health and Care Excellence (NICE) Quality and Outcomes Framework (QOF) Business Rules (https://qof.digital.nhs.uk), version 37.0 (version date 09/06/2017). Read v2 and CTV3 codes were converted to ICD-9 and ICD-10 codes using UK Biobank Resource 592 (Clinical coding classification systems and maps).

In this study, we examined all-cause dementia as well as Alzheimer's disease (AD), and vascular dementia (VaD). For those who developed dementia, time to event was the interval in years from study entry to the date of diagnosis. For those who were free of dementia, time to event was the interval in years from study entry to 31 October 2022 for those who did not have a death record, and date of death for those who were deceased. Participants who developed dementia before study entry were excluded from all analyses.

Statistical analysis. Protein levels were inverse-normal transformed to induce a Gaussian distribution, with mean 0 and standard deviation 1 for each protein. Cox's proportional hazards regression models were used to estimate the association between each protein and risk of incident dementia diagnosis, adjusted for age at assessment, sex, highest education level attained, study site, BMI, eGFR (kidney function), prevalent type-2 diabetes, and high cholesterol. Outcomes were incident all-cause dementia, Alzheimer's disease, and vascular dementia. R statistical computing platform v4.2.3 was used for all analyses, with R package {survival} v3.5-5 used to estimate time-to-event associations.

Functional characterization of *APOEε*4 resiliency-associated proteins

Gene-tissue expression analysis. Expression of genes coding for candidate proteins was examined across discrete tissue types using data from the GTEx project (version 8), a publicly available gene expression data [5]. We used the GTEx Multi Gene Query to visualize expression of candidate protein coding genes in terms of transcripts per million. Hierarchical cluster analysis was used to cluster genes and tissues based on expression. Written informed consent was provided by all donors and the GTEx protocols were approved by the NIH National Human Genome Research Institute. Tissue-specific enrichment was derived using normalized mRNA expression levels in a Human Protein Atlas and GTEX consensus dataset. A gene was considered *enriched* if the normalized transcripts per million (nTPM) in a particular tissue type is at least four times that of any other tissue type. A gene was considered *enhanced* if the nTPM of the gene in 1-5 tissues is expressed at least four times the mean of other tissues [6,7].

Cell-specific expression. Expression levels of candidate genes in brain, vascular, meningeal, and immune cells were obtained from the Human BBB (https://twc-stanford.shinyapps.io/human_bbb/),[8] a transcriptomic dataset generated using VINE (Vessel Isolation and Nuclei Extraction)-seq. We examined expression differences between AD and control brains using Welch's t test.

Ingenuity Pathway Analysis. Ingenuity Pathway Analysis (IPA) was used to characterize molecules downstream of the top three candidate proteins associated with resilient vs. non-resilient status among *APOEε*4 participants (FDR $P<0.05$). The three candidate proteins were selected using Path Builder tool and the *Grow* function was applied to define relationships between molecules (edges). We defined protein-protein relationships based on activation, causation, chemical-chemical interactions, chemical-protein interactions, inhibition, modification, molecular cleavage, phosphorylation, protein-DNA interactions, protein-protein interactions, protein-RNA interactions, regulation of binding, RNA-RNA interactions, transcription, translocation, and ubiquitination. We identified the top three canonical pathways (based on number of overlapping molecules) among the candidate proteins and downstream molecules (left) and included three canonical pathways implicated in Alzheimer's disease and related dementia (right).

Protein-protein interactions. Protein interaction networks were defined using STRING (Search Tool for the Retrieval of Interacting Genes/Proteins) (https://string-db.org), a database and visualization tool that integrates publicly available sources of information to provide a comprehensive understanding of protein–protein interaction (PPI) networks.[9] PPI clusters were derived using k-means clustering (k=4). Overrepresentation analyses were performed for each of the three protein clusters using Gene Ontology, KEGG, Reactome, and WikiPathways.

Machine learning methods for prediction of resilient vs. non-resilient status

We classified resilient vs. non-resilient status using a machine learning approach in Python 3.9 (scikit-learn package) [10]. Specifically, we utilized random forest classification (*sklearn* RandomForestClassifier). Random forest is an ensemble machine learning algorithm whereby predicted classification is determined based on an aggregation of multiple decision trees built independently and in parallel and is well-suited for classification problems with a greater number of predictors than observations [11,12]. We tested four separate classification models with the following prediction features: (a) all proteins significant at a suggestive P<0.01 threshold, (b) all proteins significant at a suggestive P<0.01 threshold and targeted ADRD biomarkers, (c) all available proteins and targeted ADRD biomarkers, and (d) age at enrollment only. Mean imputation was used for missing protein or biomarker data (ranging from 0.49-14.34%). For each tested classification model, we utilized five-fold stratified cross-validation with *GridSearch* to identify the best performing hyperparameters from a pre-determined set ('n_estimators': [100, 500, 1000, 2000], 'max_depth': [3, 5, 10, 15, None], 'max_features': ['sqrt', 'log2', None]. The random forest model with the highest AUC was selected and used for subsequent feature importance analyses. We report average values (across cross-validation folds) for accuracy, precision, recall, F1 score, and AUC for all four classification models. Feature importance was determined using Shapley Additive explanation (SHAP) values (*shap* TreeSHAP). Based on game theory, SHAP values provide an explanation of ML classification based on the sum of individual feature contributions [13].

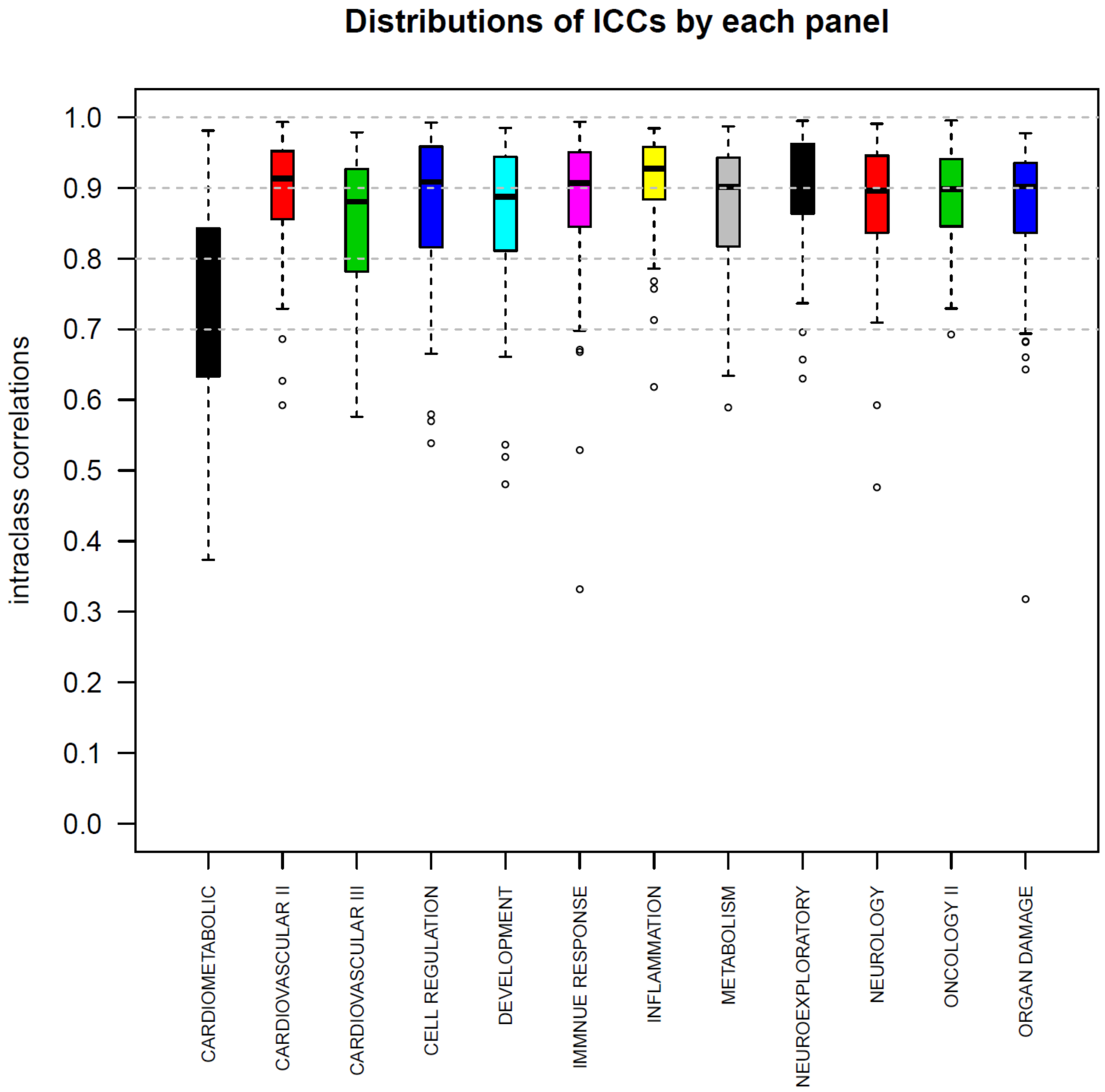

Supplementary Figure 1. Intraclass correlation for proteins on each of the 92-protein Olink panels.

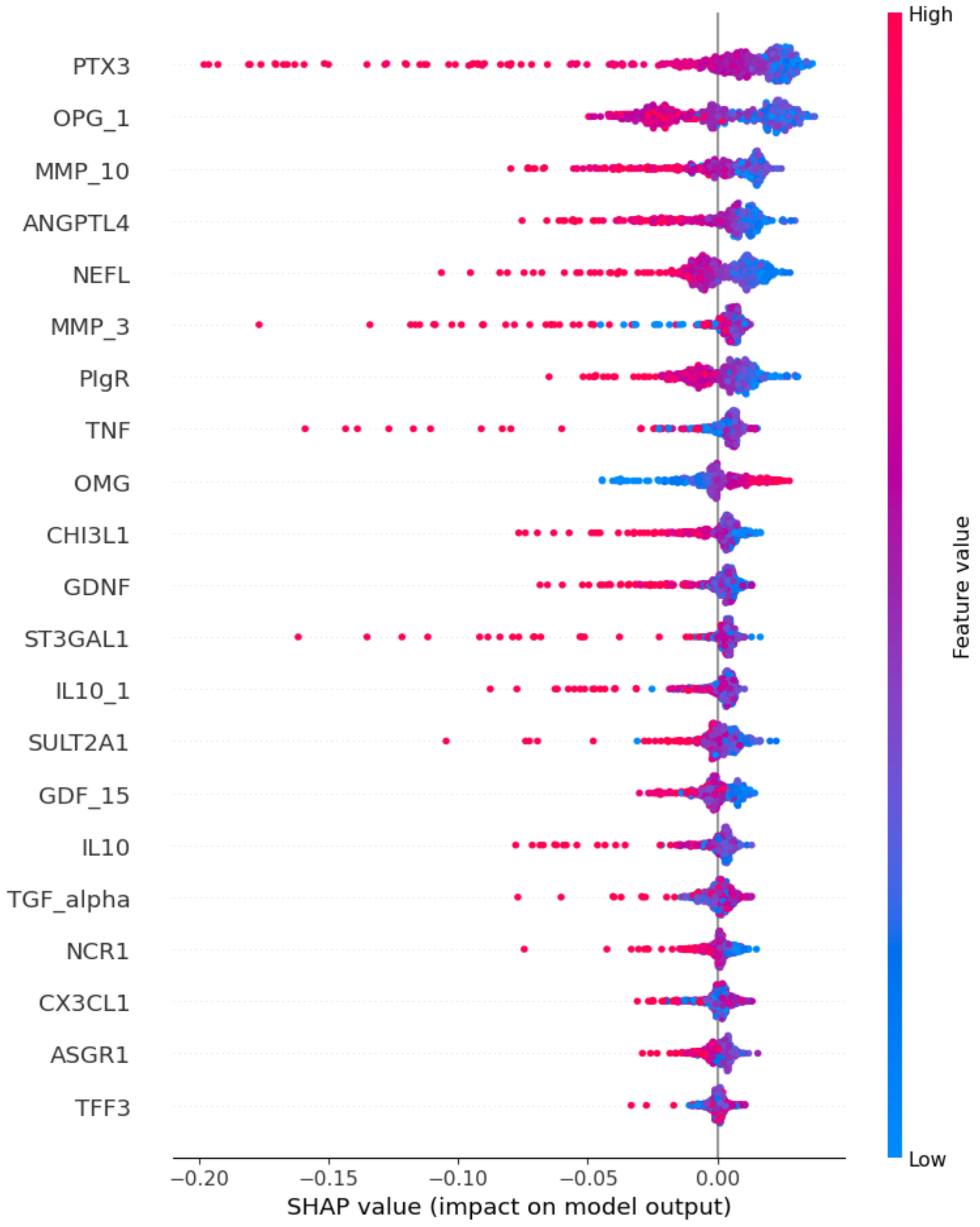

Supplementary Figure 2. Visualization of SHAP values for prediction of resilient vs. non-resilient status using all proteins significant at a suggestive *P*<0.01 threshold in the previous discovery analysis as model parameters.

Proteins are ordered top to bottom based on importance to the model, with proteins at the top being most important. A dot on the right side of the vertical line (SHAP value = 0) reflects prediction into the resilient class. The absolute SHAP value along the x-axis can be understood as the extent to which each protein contributes to the prediction of resilient vs. non-resilient status. The color of the dots in each plot reflects protein abundance for a specific participant (N=342) with red representing high protein abundance and blue representing low protein abundance. These models were derived from a random forest machine learning model.

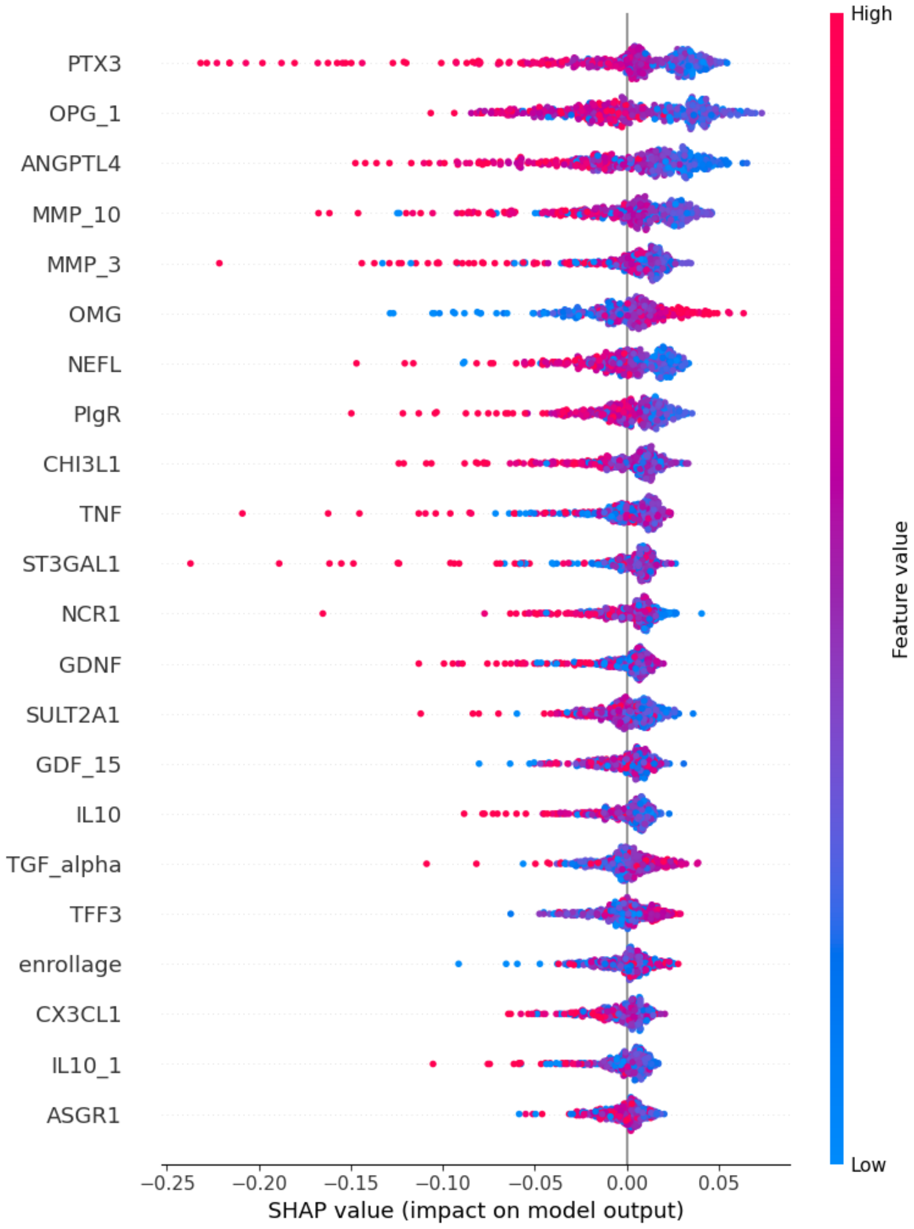

Supplementary Figure 3. Visualization of SHAP values for prediction of resilient vs. non-resilient status using all proteins significant at a suggestive *P*<0.01 threshold in the previous discovery analysis and age at study baseline as model parameters.

Proteins are ordered top to bottom based on importance to the model, with proteins at the top being most important. A dot on the right side of the vertical line (SHAP value = 0) reflects prediction into the resilient class. The absolute SHAP value along the x-axis can be understood as the extent to which each protein contributes to the prediction of resilient vs. non-resilient status. The color of the dots in each plot reflects protein abundance for a specific participant (N=342) with red representing high protein abundance and blue representing low protein abundance. These models were derived from a random forest machine learning model.

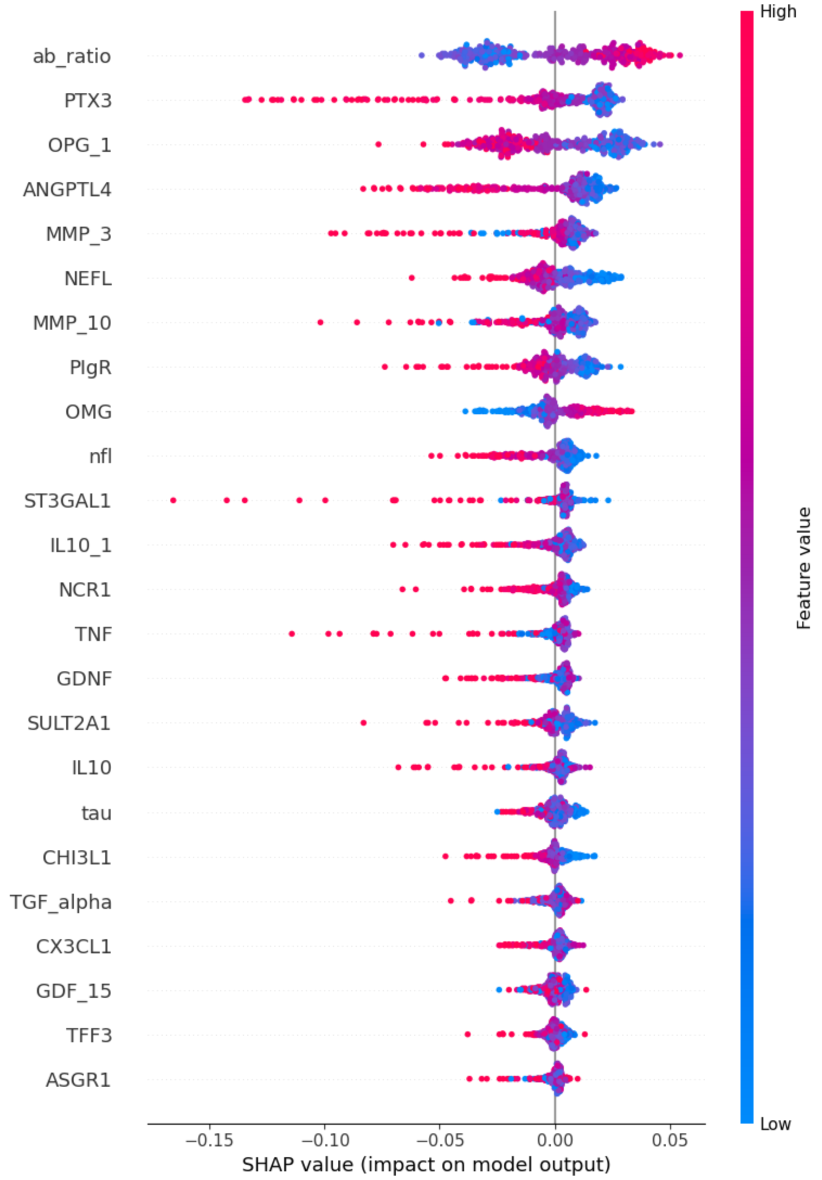

Supplementary Figure 4. Visualization of SHAP values for prediction of resilient vs. non-resilient status using all proteins significant at a suggestive *P*<0.01 threshold in the previous discovery analysis and targeted Alzheimer's disease and related dementia biomarkers ($AB_{42/40}$, total tau, NfL) as model parameters.

Proteins are ordered top to bottom based on importance to the model, with proteins at the top being most important. A dot on the right side of the vertical line (SHAP value = 0) reflects prediction into the resilient class. The absolute SHAP value along the x-axis can be understood as the extent to which each protein contributes to the prediction of resilient vs. non-resilient status. The color of the dots in each plot reflects protein abundance for a specific participant (N=342) with red representing high protein abundance and blue representing low protein abundance. These models were derived from a random forest machine learning model.

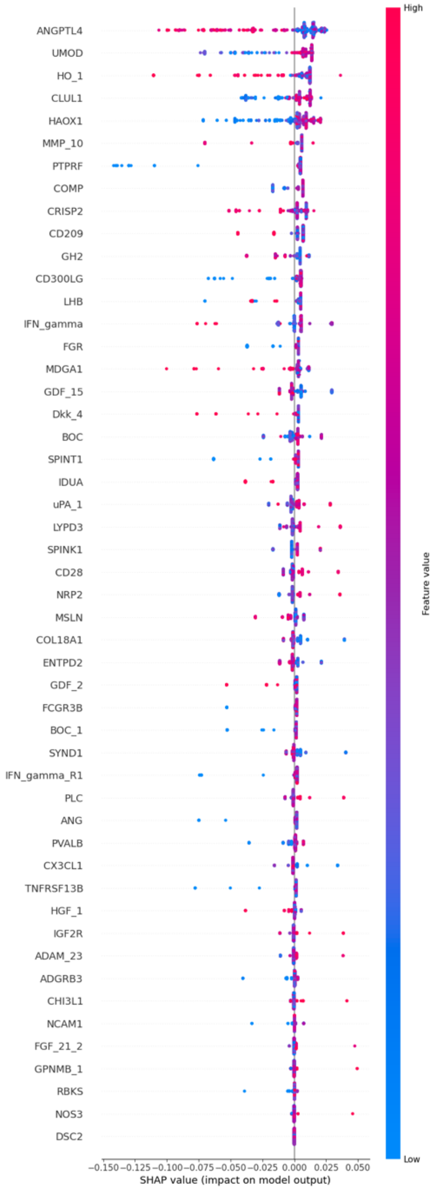

Supplementary Figure 5. Visualization of SHAP values for prediction of resilient vs. non-resilient status using all proteins collected at study baseline as model parameters.

Proteins are ordered top to bottom based on importance to the model, with proteins at the top being most important. A dot on the right side of the vertical line (SHAP value = 0) reflects prediction into the resilient class. The absolute SHAP value along the x-axis can be understood as the extent to which each protein contributes to the prediction of resilient vs. non-resilient status. The color of the dots in each plot reflects protein abundance for a specific participant (N=342) with red representing high protein abundance and blue representing low protein abundance. These models were derived from a random forest machine learning model.

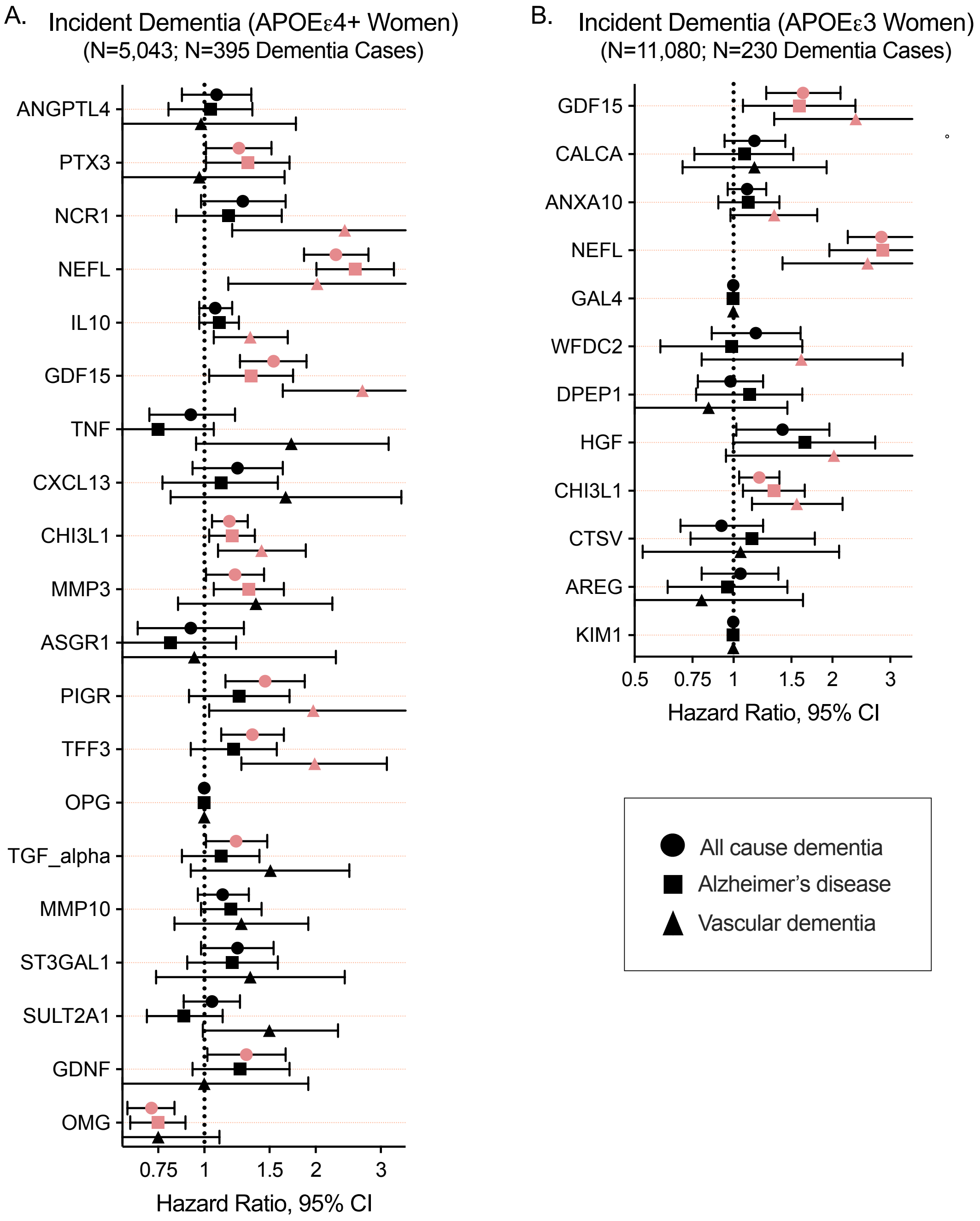

Supplementary Figure 6. Association of candidate proteins with incident all-cause, Alzheimer's, and vascular dementia among women in the UK Biobank

Association of *APOEε4* and *APOEε3* candidate proteins with incident etiology-specific dementia among female participants in the UK Biobank. Hazard ratios were derived from Cox proportional hazards models adjusted for age, sex, education study site, BMI, kidney function (eGFR), diabetes, and cholesterol. Bolded protein name indicates significant effect modification by *APOE* genotype in the discovery (WHIMS) analyses.

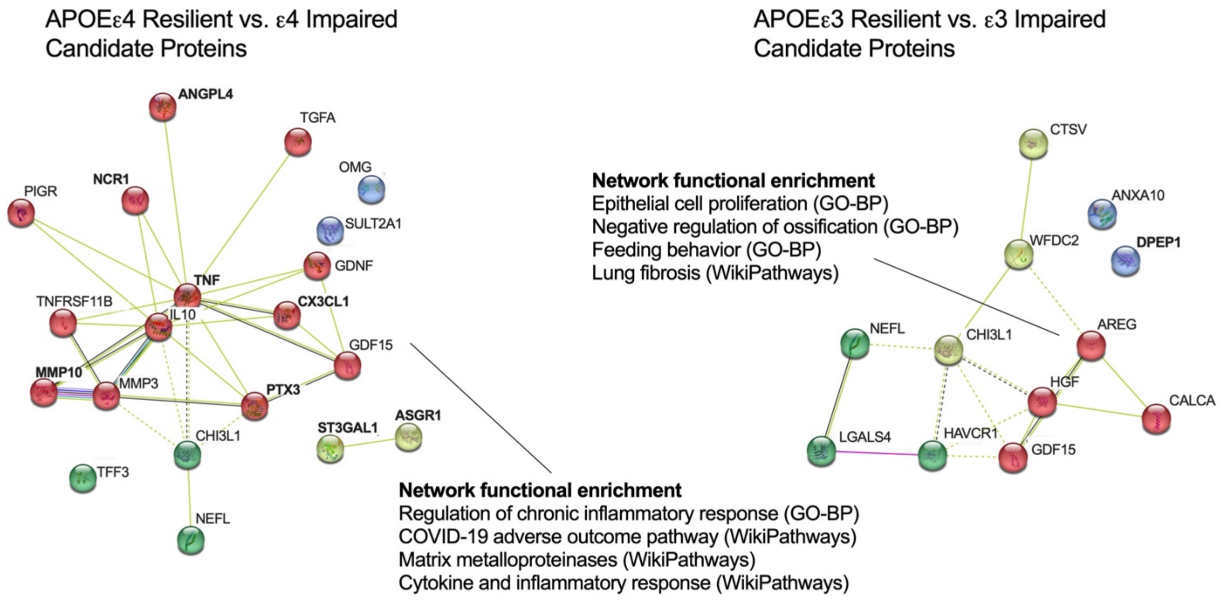

Supplementary Figure 7. Protein interaction network for proteins that significantly differed between resilient and impaired groups among *APOE*ε4 and *APOE*ε3 participants.

Using the STRING platform, proteins were clustered using K means clustering (four clusters). Functional annotation was conducted separately for each of the four sub-clusters using GO and Wiki Pathways. Functional annotation is provided for the red cluster. Bold text indicates that the association of protein with resilient vs. non-resilient status is modified by *APOE* genotype.

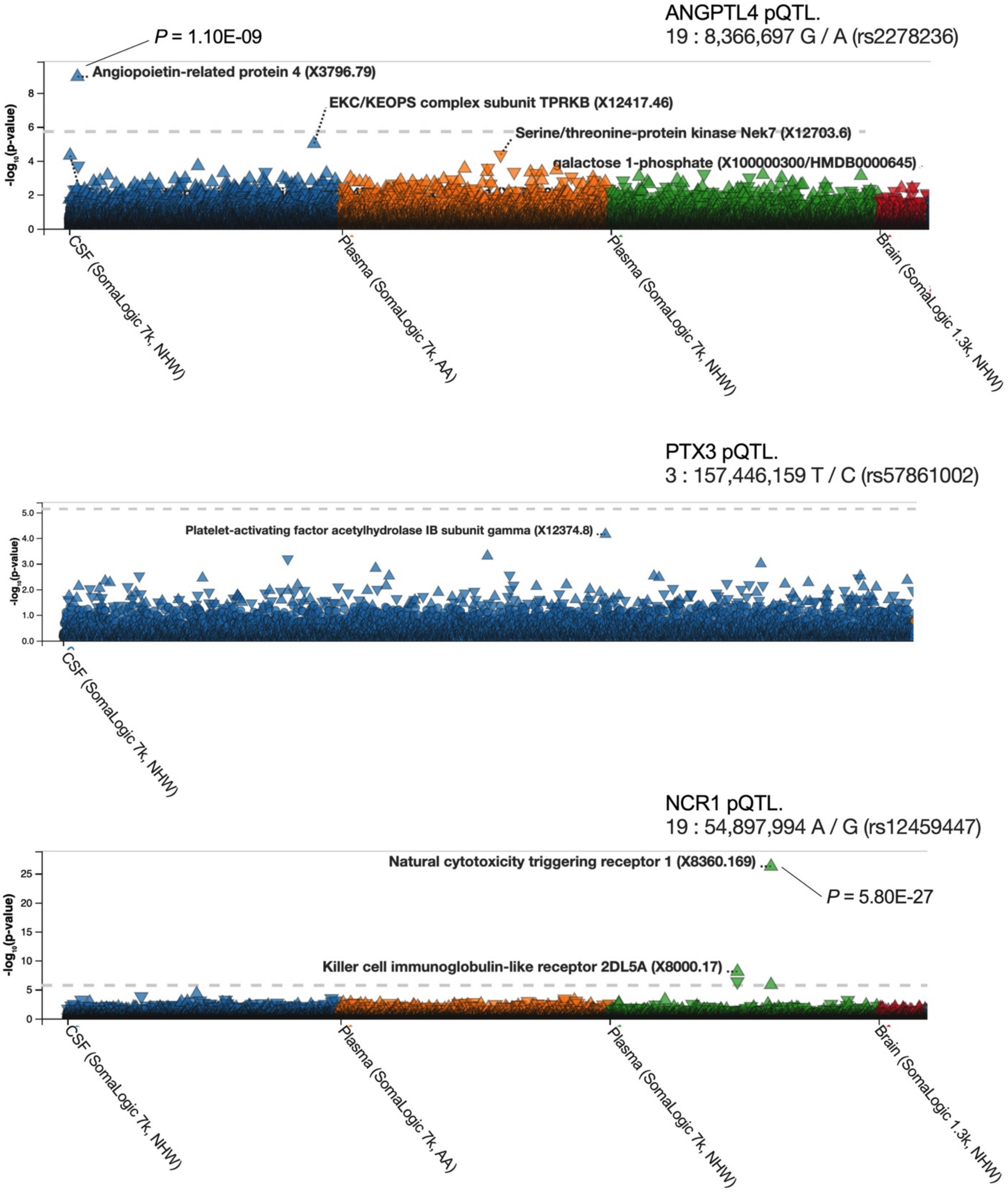

Supplementary Figure 8. Analysis of protein quantitative trait loci (pQTLs) for ANGPTL4, PTX3, and NCR1 in brain tissues, cerebral spinal fluid, and plasma.

Plasma pQTLs derived from Sun et al.[14] Data/figures derived from Online Neurodegenerative Trait Integrative Multi-Omics Explorer. https://ontime.wustl.edu/

References

1. Proteomics O. Data normalization and standardization. Olink White Pap. Olink Proteomics; 2022. p. 1–8.

2. Sun BB, Chiou J, Traylor M, Benner C, Hsu Y-HH, Richardson TG, et al. Plasma proteomic associations with genetics and health in the UK Biobank. Nature. Nature; 2023;622:329–38.

3. Gadd DA, Hillary RF, Kuncheva Z, Mangelis T, Cheng Y, Dissanayake M, et al. Blood protein levels predict leading incident diseases and mortality in UK Biobank. medRxiv. Cold Spring Harbor Laboratory Press; 2023;2023.05.01.23288879.

4. Bycroft C, Freeman C, Petkova D, Band G, Elliott LT, Sharp K, et al. The UK Biobank resource with deep phenotyping and genomic data. Nature. Nature; 2018;562:203–9.

5. Lonsdale J, Thomas J, Salvatore M, Phillips R, Lo E, Shad S, et al. The Genotype-Tissue Expression (GTEx) project. Nat. Genet. 2013. p. 580–5.

6. Uhlén M, Fagerberg L, Hallström BM, Lindskog C, Oksvold P, Mardinoglu A, et al. Tissue-based map of the human proteome. Science (80-). American Association for the Advancement of Science; 2015;347.

7. Yanai I, Benjamin H, Shmoish M, Chalifa-Caspi V, Shklar M, Ophir R, et al. Genome-wide midrange transcription profiles reveal expression level relationships in human tissue specification. Bioinformatics. Bioinformatics; 2005;21:650–9.

8. Yang AC, Vest RT, Kern F, Lee DP, Agam M, Maat CA, et al. A human brain vascular atlas reveals diverse mediators of Alzheimer's risk. Nature. Nature; 2022;603:885–92.

9. Franceschini A, Szklarczyk D, Frankild S, Kuhn M, Simonovic M, Roth A, et al. STRING v9.1: Protein-protein interaction networks, with increased coverage and integration. Nucleic Acids Res. Nucleic Acids Res; 2013;41.

10. Pedregosa FABIANPEDREGOSA F, Michel V, Grisel OLIVIERGRISEL O, Blondel M, Prettenhofer P, Weiss R, et al. Scikit-learn: Machine Learning in Python. J Mach Learn Res. 2011;12:2825–30.

11. Boateng EY, Otoo J, Abaye DA, Boateng EY, Otoo J, Abaye DA. Basic Tenets of Classification Algorithms K-Nearest-Neighbor, Support Vector Machine, Random Forest and Neural Network: A Review. J Data Anal Inf Process. Scientific Research Publishing; 2020;8:341–57.

12. Qi Y. Random Forest for Bioinformatics.

13. Marcilio WE, Eler DM. From explanations to feature selection: assessing SHAP values as feature selection mechanism. 2020 33rd SIBGRAPI Conf Graph Patterns Images. IEEE Computer Society; 2020;340–7.

14. Sun BB, Chiou J, Traylor M, Benner C, Hsu Y-H, Richardson TG, et al. Genetic regulation of the human plasma proteome in 54,306 UK Biobank participants. bioRxiv. Cold Spring Harbor Laboratory; 2022;20:2022.06.17.496443.
