# Supplementary material for: Proteomic analysis of APOEε4 carriers implicates lipid metabolism, complement and lymphocyte signaling in cognitive resilience
Source: Mol Neurodegener. 2024 Oct 31;19:81. doi: 10.1186/s13024-024-00772-2 (PMC11526661; doi:10.1186/s13024-024-00772-2)
Supplement: Supplementary file 3 — Supplementary Material 3 [file 13024_2024_772_MOESM3_ESM.pdf]

DEPARTMENT OF HEALTH & HUMAN SERVICES **Public Health Service**

National Institutes of Health
Bethesda, Maryland 20892

www.nih.gov

September 29, 2024
Guojun Bu, PhD
Editor-in-Chief
Molecular Neurodegeneration

Dear Dr. Bu and the *Molecular Neurodegeneration* Editorial Board:

On behalf of my co-authors, I would like to introduce an original research article titled "**Proteomic analysis of *APOE*ε4 carriers implicates lipid metabolism, complement and lymphocyte signaling in cognitive resilience**" for consideration for publication in *Molecular Neurodegeneration.* The reviewers responded favorably to the original manuscript and provided a number of thoughtful comments and recommendations that we have incorporated into the revised document. As you will see in the accompanying documents, we have carefully considered, responded to, and addressed each of the comments and recommendations point-by-point. We hope you will agree that the incorporation of these changes has resulted in a considerably stronger paper. Responses to reviewer comments are provided in the accompanying *Response to Reviewers* document.

Please note that we have added one author (Dr. Shannon Drouin) to revised manuscript. To address reviewer comments, Dr. Drouin performed the prediction modeling (random forest machine learning) used to determine the predictive utility of the identified proteins. She also drafted parts of the revised manuscript and the reviewer responses.

All authors have read and approved the submitted manuscript; the manuscript is not under consideration nor published elsewhere. Potential conflicts of interest, when present, are reported. As corresponding author, I take responsibility for the data, analyses, and interpretation, as well as the conduct of the research. I have had full access to the data and have the right to publish the data. For correspondence, I can be reached at:
Email: keenan.walker@nih.gov | Office Phone: 667-205-2657

Sincerely,

Keenan Walker, PhD
Tenure-Track Investigator
Multimodal Imaging of Neurodegenerative Disease (MIND) Unit
National Institute on Aging (NIA), Intramural Research Program (IRP)
